# On the use of approximate entropy and sample entropy with centre of pressure time-series

**DOI:** 10.1186/s12984-018-0465-9

**Published:** 2018-12-12

**Authors:** Luis Montesinos, Rossana Castaldo, Leandro Pecchia

**Affiliations:** 1 School of Engineering, University of Warwick, Coventry, CV4 7AL UK; 2 Escuela de Ingenieria y Ciencias, Tecnologico de Monterrey, Mexico, 14380 Mexico; 3 Institute of Advanced Study, University of Warwick, Coventry, CV4 7HS UK

**Keywords:** Approximate entropy, Sample entropy, Human balance, Postural control, Posturography, Centre of pressure

## Abstract

**Background:**

Approximate entropy (ApEn) and sample entropy (SampEn) have been previously used to quantify the regularity in centre of pressure (COP) time-series in different experimental groups and/or conditions. ApEn and SampEn are very sensitive to their input parameters: *m* (subseries length), *r* (tolerance) and *N* (data length). Yet, the effects of changing those parameters have been scarcely investigated in the analysis of COP time-series. This study aimed to investigate the effects of changing parameters *m*, *r* and *N* on ApEn and SampEn values in COP time-series, as well as the ability of these entropy measures to discriminate between groups.

**Methods:**

A public dataset of COP time-series was used. ApEn and SampEn were calculated for *m* = {2, 3, 4, 5}, *r* = {0.1, 0.15, 0.2, 0.25, 0.3, 0.35, 0.4, 0.45, 0.5} and *N* = {600, 1200} (30 and 60 s, respectively). Subjects were stratified in young adults (age < 60, *n* = 85), and older adults (age ≥ 60) with (*n* = 18) and without (*n* = 56) falls in the last year. The effects of changing parameters *m*, *r* and *N* on ApEn and SampEn were investigated with a three-way ANOVA. The ability of ApEn and SampEn to discriminate between groups was investigated with a mixed ANOVA (within-subject factors: *m*, *r* and *N*; between-subject factor: group). Specific combinations of *m*, *r* and *N* producing significant differences between groups were identified using the Tukey’s honest significant difference procedure.

**Results:**

A significant three-way interaction between *m*, *r* and *N* confirmed the sensitivity of ApEn and SampEn to the input parameters. SampEn showed a higher consistency and ability to discriminate between groups than ApEn. Significant differences between groups were mostly observed in longer (*N* = 1200) COP time-series in the anterior-posterior direction. Those differences were observed for specific combinations of *m* and *r*, highlighting the importance of an adequate selection of input parameters.

**Conclusions:**

Future studies should favour SampEn over ApEn and longer time-series (≥ 60 s) over shorter ones (e.g. 30 s). The use of parameter combinations such as SampEn (m = {4, 5}, r = {0.25, 0.3, 0.35}) is recommended.

**Electronic supplementary material:**

The online version of this article (10.1186/s12984-018-0465-9) contains supplementary material, which is available to authorized users.

## Background

Human balance is the result of the complex integration of several sensorimotor control systems (namely, the visual, vestibular, somatosensory and musculoskeletal systems) [1]. Normal ageing, specific pathologies and transient factors (e.g., loss of visual acuity, rheumatoid arthritis and orthostatic hypotension, respectively) may impair one or more of those systems [2, 3]. These impairments produce a balance deficit, increasing the risk of falling and its consequences (e.g. mild to severe or even fatal injuries, such as hip fracture and head trauma) [4]. Therefore, the characterisation of human balance in both healthy and pathological populations has been drawing the attention of researchers and clinicians alike for the last few decades [5–8].

One of the most common techniques to measure human balance is static posturography (a.k.a. stabilometry), which is the measure of the centre of pressure (COP) displacement during quiet standing. The COP is the point of application of the vertical ground reaction force vector and represents a weighted average of all the pressures over the surface of the area in contact with the ground [1]. It is typically acquired with a force platform which produces a two-dimensional time-series representing the COP trajectory in the anterior-posterior (AP) and medial-lateral (ML) axes. COP excursions are characterised by computing a number of measures. Traditionally, linear and frequency measures have been used for this purpose (e.g. total length of the COP displacement and range in the AP/ML direction, mean and median frequencies, among others) [5–8].

More recently, entropy measures have been used to assess the regularity or predictability within COP time-series collected under different testing conditions and/or in different experimental groups [9–24]. A regular, therefore more predictable process produces lower entropy values than a less regular one [25, 26]. Two commonly used methods are approximate entropy (ApEn) and sample entropy (SampEn). For instance, Cavanaugh et al. used ApEn to evaluate the effect of performing a secondary cognitive task on postural control in a sample of healthy young adults (*n* = 30), as compared to performing a single task (i.e., posture control plus cognitive task versus posture control only) [11]. The authors observed generally higher ApEn values in the anterior-posterior COP time-series during dual-task than during a single task. However, no significant differences in ApEn values for the medial-lateral direction were observed. In another study, Borg and Laxåback investigated the differences in SampEn values between young adults (*n* = 45) and older adults (*n* = 91) [17]. Significant differences between groups were observed for the AP axis with higher values for older adults than for young adults. Moreover, for the older adults higher SampEn values were observed in the AP axis than in the ML axis, whereas in general no significant AP-ML differences were observed in the young adults group.

Generally speaking, given a time-series of length *N*, ApEn(*m*, *r*, *N*) is approximately equal to the negative average natural logarithm of the conditional probability that two subseries of length *m* that are similar (within a tolerance given by ±*r* times the standard deviation of the time-series) remain similar for subseries of length *m* + 1. ApEn generates a unit-less number from 0 to 2: an ApEn value equal to zero corresponds to a time-series that is perfectly regular (e.g. a periodic signal), whereas an ApEn value equal to 2 is produced by random time-series (e.g. Gaussian noise) [25]. Importantly, the ApEn algorithm counts each subseries as matching itself. As a consequence, the ApEn algorithm inherently produces a bias towards regularity. In order to counteract this shortcoming, the SampEn algorithm does not count self-matches. SampEn(*m*, *r*, *N*) is the negative natural logarithm of the conditional probability that two subseries similar for *m* points remain similar for *m* + 1, where self-matches are not included in calculating the probability. In addition to eliminating self-matches, it has been shown that SampEn is largely independent of the data length and shows more consistent behaviours than ApEn [26].

The appropriate selection of parameters *m* (subseries length), *r* (similarity tolerance) and *N* (data length) is critical. Traditionally, for clinical data, *m* is to be set at 2 or 3, *r* is to be set between 0.1 and 0.25 times the standard deviation of the data and *N* as equal to or greater than 1000 [25, 26]. However, these recommendations were based on the analysis of cardiac and respiratory time-series, thus do not always produce optimal results for all types of data. Therefore, an investigation of the effects of changing parameter values on the computation of ApEn and SampEn for specific types of data is needed. A previous study addressed this issue in the context of spatiotemporal gait measures analysis (i.e. step length, step width and step time) [27]. However, the issue has not been investigated in a systematic manner when dealing with COP time-series.

The aims of this study were (1) to examine the effect of changing the value of parameters *m*, *r* and *N* on ApEn and SampEn values in COP time-series, and (2) to determine the ability of ApEn and SampEn to discriminate between experimental groups. To do this, seventy-two different combinations of parameter values *m*, *r* and *N* were used to calculate ApEn and SampEn values from COP time-series. The COP time-series were taken from a public dataset of posturography data from 163 participants, which were stratified in 3 groups: young adults, older adults without falls in the last 12 months and older adults with at least one fall in the last 12 months. It was expected that the values of ApEn and SampEn would change significantly as a function of *m*, *r* and *N*. However, it was foreseen that SampEn would exhibit a more consistent behaviour than ApEn across different parameter value combinations, a presumption based on previous studies using ApEn and SampEn for the analysis of inter-beat intervals, electroencephalographic signals and gait measures time-series [26–28]. Furthermore, it was expected to observe a significant difference in entropy values between young and older adults (non-fallers and fallers), in line with previous findings [13, 17]. Moreover, it was postulated that significant differences in entropy values between non-fallers and fallers would be observed for some combinations of parameter values *m*, *r* and *N*.

The present study was motivated by the promising results obtained in a preliminary study, in which the issue of the adequate selection of ApEn and SampEn parameter values for COP time-series analysis was partially addressed [29]. However, the present study represents a more comprehensive investigation of this issue, as it covered a wider range of parameter values, included the ML direction in addition to the AP direction and compared the ability of ApEn and SampEn to discriminate between experimental groups to that of traditional measures of COP displacement (e.g. total length). Therefore, we consider the methods followed in the present study to be more robust and the results to be more informative for future studies. Importantly, despite the weaknesses that ApEn has compared to SampEn, it has already shown to be more sensitive than traditional measures for detecting altered postural control after mild traumatic brain injury [10, 18] and the effects of a secondary cognitive task on postural control [11]. Hence, we decided to adopt an open stance towards the performance of ApEn, rather than generalising conclusions supported on the analysis of other types of biological time-series, such as inter-beat intervals, EEG and gait measures [26–28].

## Methods

### Dataset description

The present study made use of a public dataset of human balance evaluations [30]. This dataset contains posturography data from 163 participants. A detailed description of the protocol, the data pre-processing methods and the resulting dataset can be found in [31]. Briefly, COP time-series were recorded while subjects were standing still for 60 s in four different conditions: with eyes open on a rigid surface, with eyes open on a foam mat, with eyes closed on a rigid surface, and with eyes closed on a foam mat. Three trials per condition were recorded, producing 1930 trials in total (the authors reported 26 trials from 5 subjects as missing due to the inability of those subjects to complete the tasks). During the trials, 3D ground reaction forces and moments were recorded using a force platform with a sampling frequency of 100 Hz and were later used to compute the COP position in the anterior-posterior and medial-lateral axes. Importantly, the authors of this dataset reported having smoothed the signals using a 4th-order zero-lag Butterworth low-pass filter with a cut-off frequency of 10 Hz. Previous studies have investigated the effects of digital filtering (specifically using a 2nd-order dual-pass Butterworth low-pass filter) on traditional and entropy measures of COP displacement (standard deviation/RMS value and sample entropy, respectively) [32, 33]. While digital filtering had no effect on traditional measures [32], a decrease in sample entropy was reported for filtered data compared to unfiltered data [32, 33]. Should our data analysis be replicated on unfiltered COP data, higher entropy values would be expected to come out from it.

Additionally, this public dataset contains basic demographic, anthropometric, and health status data for each participant (e.g. age, height, weight, morbidities and disabilities), as well as their scores for other evaluations related to balance, fear of falling, physical activity and cognitive function.

### Data processing

Besides *m*, *r* and *N*, the ApEn and SampEn algorithms allow adjusting a fourth parameter known as time delay (τ) in the computation of entropy values. Generally speaking, by adjusting the time delay to a specific value of τ, the time-series used for the computation of ApEn/SampEn would be made of the first sample and then every τth sample after the first. In more formal terms, for a time-series *X* of length *N*, *X* = {x(1), x(2), x(3), …, x(*N*)}, the computation of ApEn/SampEn with a time delay of τ would be performed on the time-series given by *X’* = {x(1), x(1 + τ), x(1 + 2τ), …, X(*N*-τ + 1)}. In a previous study, Kaffashi et al. [34] showed that, for time-series generated by non-linear dynamics that have a long-range autocorrelation (e.g. a slowly decaying autocorrelation function or ACF, such as the observed for COP time-series), using a unity delay (τ = 1) would solely measure the linear autocorrelation properties of the signal. This would mask the ability of ApEn/SampEn to quantify the regularity in the time-series resulting from long-range non-linear features. For this type of data, then, using a higher time-delay value was suggested. Therefore, in our study, COP time-series were downsampled by a factor of 5, indirectly adjusting the time delay (τ = 5) in the computation of ApEn and SampEn [34]. Consequently, the downsampled data had an effective frequency of 20 Hz, resulting in a length of *N* = 1200 data points (20 Hz × 60 s).

To examine the effect of the choice of input parameters *m*, *r* and *N*, each COP time-series was subjected to ApEn and SampEn calculation for all possible combinations of *m* = {2, 3, 4, 5}, *r* = {0.1, 0.15, 0.2, 0.25, 0.3, 0.35, 0.4, 0.45, 0.5} and *N* = {600, 1200} (i.e., 30 and 60 s, respectively). These ranges of input parameter values are wider than the ones adopted in previous studies, in which values of *m* equal to 2, 3 or 5 and *r* from 0.1 to 0.3 have been used [9–12, 14–18, 22–24]. This choice was motivated by our interest in exploring the behaviour of ApEn and SampEn for a range of input parameters extending beyond the traditional values. A detailed description of the algorithms used to compute ApEn and SampEn can be found in [25, 26], respectively.

Additionally, COP displacement linear measures were also computed as described in [6]: total length of displacement, amplitude of displacement in the AP and ML axes, standard deviation in the AP and ML axes, mean velocity in the AP and ML axes, total mean velocity and area covered by the displacement. These measures were only computed for COP time-series of length *N* = 1200 (i.e. 60 s).

The scripts for data processing were written in MATLAB R2017b (The Mathworks, Inc., Natick, MA, USA).

### Statistical analyses

#### Effects of changing m, r and N parameters on ApEn and SampEn

A three-way ANOVA was conducted to determine the effect of changing *m*, *r* and *N* on ApEn and SampEn values. As described before, there were four levels of *m* (i.e., 2, 3, 4, 5), nine levels of *r* (i.e. 0.1, 0.15… 0.5) and two levels of *N* (i.e. 600 and 1200). A significant three-way interaction between *m*, *r*, and *N* (*p*-value < 0.05) indicated that entropy values were changing significantly for one or more combinations of *m*, *r* and *N*. Otherwise, a significant two-way interaction indicated that entropy values were changing significantly for one or more combinations of those two parameters, yet entropy values were not significantly different across the values of the third parameter. These analyses were performed including ApEn and SampEn values for all COP time-series, regardless of testing condition (i.e. all trials per testing condition were included).

#### Ability of ApEn and SampEn to discriminate between experimental groups

Firstly, subjects were stratified in three groups based on their age and history of falls in the past 12 months: young adults (Young, age < 60), older adults (age ≥ 60) without falls in the last 12 months (Non-Fallers) and older adults (age ≥ 60) who experienced one or more falls in the last 12 months (Fallers). Subjects with reported physical disabilities were excluded from the analysis.

Subsequently, ApEn and SampEn group mean and standard deviation values by group for all combinations of *m*, *r* and *N* were computed, regardless of testing condition. To determine the effects of group on ApEn and SampEn a mixed-design ANOVA was conducted. It consisted of one between-subjects factor (i.e., group) and three within-subjects factors (i.e., *m*, *r* and *N*). There were three levels for group (i.e. Young, Non-Fallers and Fallers); the levels for the within-subject factors have been introduced above. A significant four-way interaction between group, *m*, *r* and *N* indicated that the entropy values were different between at least two groups for one or more combinations of *m*, *r* and *N*. Those combinations were identified by performing a post hoc analysis of the differences between groups for each combination of *m*, *r* and *N* using the Tukey’s honest significant difference procedure. A *p*-value < 0.05 was accepted as evidence of statistical significance. Moreover, the statistical significance of differences in linear measures between groups was also determined using a one-way ANOVA and a post hoc analysis (Tukey’s honest significant difference procedure). These later analyses were performed in order to compare the ability of ApEn and SampEn to discriminate between different groups to that of the standard methods.

#### Behaviour of SampEn in different testing conditions

Additionally, the behaviour of SampEn in different testing conditions was also investigated. Namely, SampEn mean and standard deviation values by group for all combinations of *m*, *r* and *N* were computed separately for each testing condition: eyes open on a rigid surface (OR), eyes closed on a rigid surface (CR), eyes open on a foam mat (OF) and eyes closed on a foam mat (CF). For each testing condition, a one-way ANOVA with group as factor, as well as a post hoc analysis (Tukey’s honest significant difference), were performed for each parameter combination. These analyses were carried out in order to determine whether a specific testing condition might boost the sensitivity of SampEn to differences between groups (e.g. more parameter combinations produced significant differences between groups). These analyses were performed only on SampEn values from COP time-series in the anterior-posterior direction, as the analyses described earlier revealed that these were more sensitive to differences between groups, especially between Non-Fallers and Fallers.

All statistical analyses were performed in MATLAB R2017b.

## Results

### Effects of changing *m*, *r* and *N* parameters on ApEn and SampEn

For ApEn in the anterior-posterior direction, a three-way ANOVA with *m*, *r*, and *N* as factors revealed a main effects of *m* [F(3, 138888) = 8195, *p* < 0.001, partial η^2^ = 0.15], *r* [F(8, 138888) = 19467, *p* < 0.001, partial η^2^ = 0.53] and *N* [F(1, 138888) = 25.8, *p* < 0.001, partial η^2^ = 0.0002]. These main effects were qualified by an interaction between *m*, *r* and *N* [F(24, 138888) = 12. 8, *p* < 0.001, partial η^2^ = 0.002]. For ApEn in the medial-lateral direction, the three-way ANOVA revealed a main effects of *m* [F(3, 138888) = 13287, *p* < 0.001, partial η^2^ = 0.22], *r* [F(8, 138888) = 28481, *p* < 0.001, partial η^2^ = 0.62] and *N* [F(1, 138888) = 183.8, *p* < 0.001, partial η^2^ = 0.001]. These main effects were qualified by an interaction between *m*, *r* and *N* [F(24, 138888) = 14.9, *p* < 0.001, partial η^2^ = 0.003]. The presence of significant three-ways interactions suggests that ApEn values were significantly changing for different combinations of *m*, *r* and *N*.

For SampEn in the anterior-posterior direction, a three-way ANOVA with *m*, *r*, and *N* as factors revealed a main effects of *m* [F(3, 138,888) = 1,195, *p* < 0.001, partial η^2^ = 0.025], *r* [F(8, 138,888) = 25,612, *p* < 0.001, partial η^2^ = 0.6] and *N* [F(1, 138888) = 1416, *p* < 0.001, partial η^2^ = 0.01]. These main effects were qualified by interactions between *m* and *r* [F(24, 138888) = 69.4, *p* < 0.001, partial η^2^ = 0.012], between *m* and *N* [F(3, 138888) = 19.6, *p* < 0.001, partial η^2^ = 0.0004] and between *r* and *N* [F(8, 138888) = 3.78, *p* < 0.001, partial η^2^ = 0.0002]. The interaction between *m*, *r*, and *N* was not significant [F(24, 138888) = 0.28, *p* = 0.99, partial η^2^ = 0]. For SampEn in the medial-lateral direction, the three-way ANOVA revealed a main effects of *m* [F(3, 138888) = 2561, *p* <, partial η^2^ = 0.052], *r* [F(8, 138888) = 35595, *p* < 0.001, partial η^2^ = 0.67] and *N* [F(1, 138888) = 2719, p < 0.001, partial η^2^ = 0.019]. These main effects were qualified by interactions between *m* and *r* [F(24, 138888) = 153.3, *p* < 0.001, partial η^2^ = 0.026), *m* and *N* [F(24, 138888) = 40.6, *p* < 0.001, partial η^2^ = 0.0009) and *r* and *N* (F(24, 138888) = 6.74, *p* < 0.001, partial η^2^ = 0.0004). The interaction between m, r, and N was not significant [F(24, 138888) = 0.53, *p* = 0.97, partial η^2^ = 0.0001). The presence of significant two-way interactions suggests that SampEn values were changing significantly for one or more combinations of those two parameters, yet entropy values were not significantly different across the values of the third parameter.

**Fig. 1:**
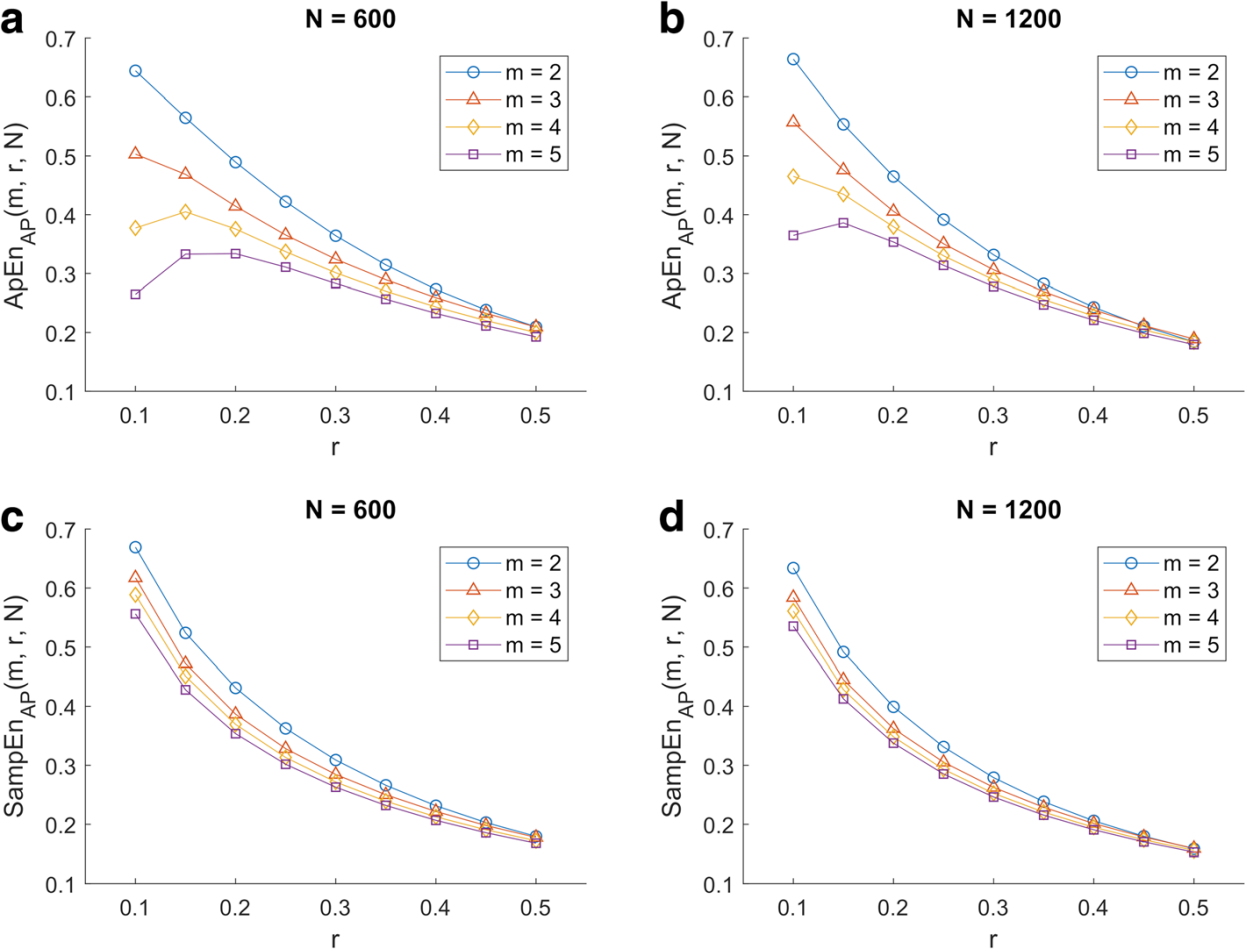
Approximate Entropy (ApEn) and Sample Entropy (SampEn) as a function of *m*, *r* and *N* for the anterior-posterior (AP) component of the centre of pressure displacement during quiet standing: **a**) ApEn for *N* = 600, **b**) ApEn for *N* = 1200, **c**) SampEn for *N* = 600, and **d**) SampEn for *N* = 1200

These findings are illustrated in Fig. 1, where ApEn and SampEn for the AP component are presented as a function of *m*, *r* and *N*. It can be observed that the shape of ApEn as a function of *r* was different for different combinations of *m* and *N* (top panels). As for SampEn, its values tended to decrease as *r* increased, yet its shape was consistent across different combinations of *m* and *N* (bottom panels). Both ApEn and SampEn showed a similar behaviour for the medial-lateral component of the COP displacement (Additional file 1:Figure S1).

### Ability of ApEn and SampEn to discriminate between experimental groups

#### Participant grouping and characteristics

Four participants were discarded from this analysis due to physical disabilities (namely, poliomyelitis and cerebral palsy), leaving 159 participants (115 females, 44 males) for the analysis: 85 subjects were young adults (Young), 56 subjects were older adults without falls in the last 12 months (Non-Fallers) and 18 subjects were older adults one or more falls in the last 12 months (Fallers). Table 1 shows the mean value (standard deviation) for participant characteristics by group: age, height, weight and body mass index (BMI). Moreover, it shows results from a one-way ANOVA and post hoc comparisons between groups carried out using the Tukey’s honest significant difference procedure. No significant differences were observed between the Non-Fallers and Fallers groups, suggesting homogeneity between them with regards to age and basic anthropometric variables (thus discarding those characteristics as potential confounders).

**Table 1:** Participant characteristics by group

	One-way ANOVA		Descriptive statistics by group			Post hoc					
			Young (*n* = 85)	Non-Fallers (*n* = 56)	Fallers (*n* = 18)	NF - Y		F - Y		F - NF	
Variable	F	*p*-value	Mean(SD)	Mean(SD)	Mean(SD)	MD	*p*-value	MD	*P*-value	MD	*p*-value
Age, years	722.3	< 0.001	27.7(7.78)	71.5(6.35)	71.2(7.12)	43.8	**< 0.001**	43.5	**<0.001**	−0.3	0.984
Height, cm	26.2	< 0.001	166.8(8.75)	157.8(8.73)	155.2(6.16)	−9	**< 0.001**	-11.6	**<0.001**	−2.6	0.502
Weight, kg	2.24	0.11	61.6(7.73)	63.9(8.43)	60(8.10)	2.3	0.207	-1.6	0.718	−3.9	0.163
BMI, kg/m^2^	26.7	< 0.001	22.2(2.82)	25.7(2.97)	24.9(2.84)	3.5	**< 0.001**	2.7	**0.001**	−0.8	0.540

*F* F = statistic deviation, *MD* mean difference, *BMI* body mass index. Bold values indicate significant differences

#### Approximate entropy

A significant four-way interaction between group, *m*, *r* and *N* was found [Anterior-Posterior: F(6.96, 6601) = 16.3, *p* < 0.001, partial η^2^ = 0.17; Medial-Lateral: F(6.99, 6624) = 5.43, *p* < 0.001, partial η^2^ = 0.006]. This indicated that the ApEn values were different between at least two groups for one or more combinations of *m*, *r* and *N*. Importantly, the reported *p*-values are the corrected ones using the Greenhouse-Geisser procedure, given that the compound symmetry assumption was violated (Mauchly’s test with a *p* < 0.001 for both anterior-posterior and medial-lateral COP time-series).

##### Young versus Older adults (Non-Fallers and Fallers)

**Fig. 2:**
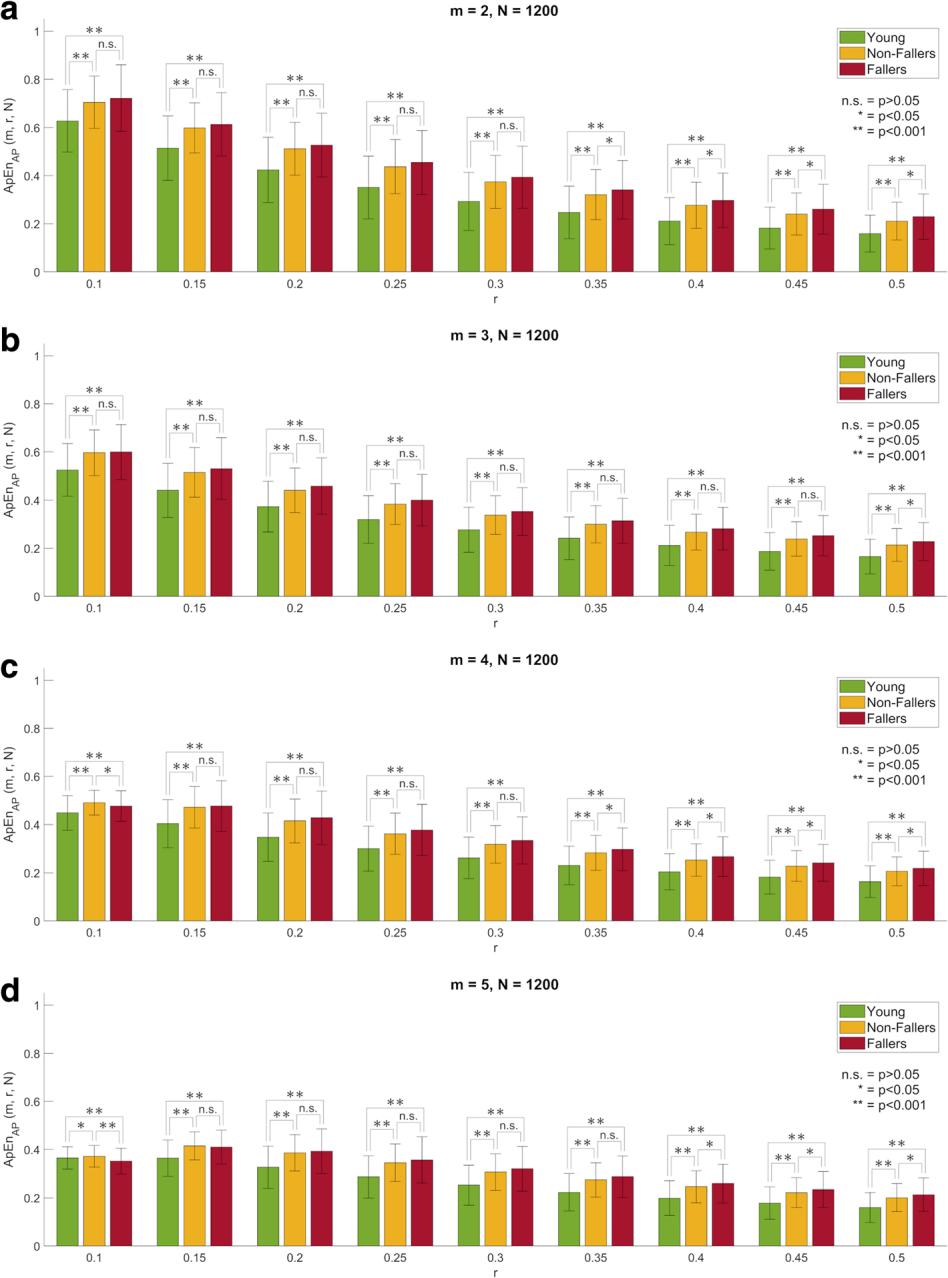
Approximate entropy (ApEn) mean value (bars) and standard deviation (error lines) by group as a function of *r* for *m* = {2, 3, 4, 5} (from top to bottom) and *N* = 1200 (i.e. 60 s) for the anterior-posterior (AP) component of the centre of pressure displacement during quiet standing: **a**) *m* = 2, **b**) *m* = 3, **c**) *m* = 4, and **d**) *m* = 5

For *N* = 1200 (i.e. 60 s) in the AP direction, Fallers and Non-Fallers showed generally higher ApEn mean values than Young adults (Fig. 2). There was only one exception to this trend (namely, for ApEn(*m* = 5, *r* = 0.1)) for which Fallers had a slightly lower ApEn mean value than Young adults (a behaviour hereon referred to as “trend flip” or “crossover”). Statistical testing revealed that those differences were significant (*p* < 0.05) for all combinations of *m* and *r* (Table 2). In the ML direction, Fallers had lower ApEn mean values than Young adults for all combinations of *m* and *r* (Additional file 1: Figure S2). However, statistical testing revealed that only one combination of *m* and *r* produced significant differences between groups (Additional file 2: Table S2). The differences between Young adults and Non-Fallers did not exhibit a consistent trend.

**Table 2:** Approximate entropy in the anterior-posterior direction as a function of *r* and *m* for a data length of *N*=1200 (i.e. 60-seconds)

	One-way ANOVA		Descriptive statistics by group						Post-hoc					
			Young (Y)		Non-Fallers (NF)		Fallers (F)		NF - Y		F - Y		F - NF	
*r*	F	*p*-value	Mean	SD	Mean	SD	Mean	SD	MD	*p*-value	MD	*p*-value	MD	*p*-value
*m = 2*														
0.1	105.45	**<0.001**	0.628	0.129	0.705	0.108	0.723	0.138	0.078	**<0.001**	0.095	**<0.001**	0.017	0.178
0.15	119.31	**<0.001**	0.515	0.134	0.599	0.104	0.613	0.131	0.084	**<0.001**	0.099	**<0.001**	0.015	0.298
0.2	124.58	**<0.001**	0.424	0.136	0.512	0.110	0.527	0.133	0.088	**<0.001**	0.103	**<0.001**	0.015	0.280
0.25	126.90	**<0.001**	0.351	0.131	0.438	0.113	0.455	0.133	0.087	**<0.001**	0.104	**<0.001**	0.018	0.176
0.3	126.80	**<0.001**	0.293	0.121	0.374	0.110	0.394	0.129	0.081	**<0.001**	0.101	**<0.001**	0.019	0.093
0.35	125.88	**<0.001**	0.247	0.109	0.321	0.104	0.341	0.122	0.074	**<0.001**	0.094	**<0.001**	0.020	**0.049**
0.4	124.51	**<0.001**	0.211	0.098	0.277	0.096	0.297	0.113	0.066	**<0.001**	0.086	**<0.001**	0.020	**0.024**
0.45	123.46	**<0.001**	0.182	0.087	0.241	0.087	0.260	0.104	0.059	**<0.001**	0.078	**<0.001**	0.020	**0.014**
0.5	122.47	**<0.001**	0.159	0.077	0.211	0.079	0.229	0.094	0.052	**<0.001**	0.070	**<0.001**	0.019	**0.009**
*m = 3*														
0.1	111.29	**<0.001**	0.526	0.109	0.597	0.094	0.600	0.114	0.071	**<0.001**	0.074	**<0.001**	0.003	0.939
0.15	118.46	**<0.001**	0.441	0.113	0.516	0.103	0.531	0.128	0.074	**<0.001**	0.090	**<0.001**	0.016	0.170
0.2	117.94	**<0.001**	0.374	0.106	0.441	0.093	0.459	0.117	0.068	**<0.001**	0.085	**<0.001**	0.017	0.080
0.25	121.98	**<0.001**	0.320	0.099	0.384	0.085	0.400	0.107	0.064	**<0.001**	0.080	**<0.001**	0.016	0.073
0.3	123.27	**<0.001**	0.277	0.094	0.338	0.081	0.353	0.099	0.061	**<0.001**	0.076	**<0.001**	0.015	0.088
0.35	125.37	**<0.001**	0.242	0.089	0.300	0.078	0.314	0.094	0.058	**<0.001**	0.073	**<0.001**	0.014	0.084
0.4	125.87	**<0.001**	0.212	0.084	0.267	0.075	0.282	0.089	0.055	**<0.001**	0.070	**<0.001**	0.014	0.064
0.45	125.41	**<0.001**	0.187	0.078	0.239	0.072	0.253	0.084	0.052	**<0.001**	0.066	**<0.001**	0.014	0.057
0.5	124.92	**<0.001**	0.166	0.073	0.214	0.068	0.228	0.079	0.048	**<0.001**	0.062	**<0.001**	0.014	**0.043**
*m = 4*														
0.1	88.78	**<0.001**	0.449	0.072	0.491	0.052	0.477	0.064	0.042	**<0.001**	0.028	**<0.001**	-0.014	**0.013**
0.15	124.48	**<0.001**	0.404	0.099	0.472	0.086	0.477	0.105	0.068	**<0.001**	0.073	**<0.001**	0.005	0.785
0.2	121.79	**<0.001**	0.348	0.100	0.415	0.091	0.428	0.111	0.067	**<0.001**	0.080	**<0.001**	0.013	0.203
0.25	121.37	**<0.001**	0.301	0.094	0.362	0.085	0.378	0.106	0.062	**<0.001**	0.077	**<0.001**	0.016	0.075
0.3	121.01	**<0.001**	0.262	0.087	0.319	0.078	0.334	0.097	0.057	**<0.001**	0.072	**<0.001**	0.015	0.054
0.35	122.14	**<0.001**	0.231	0.080	0.283	0.073	0.298	0.089	0.052	**<0.001**	0.067	**<0.001**	0.015	**0.045**
0.4	123.08	**<0.001**	0.205	0.075	0.254	0.068	0.268	0.082	0.049	**<0.001**	0.063	**<0.001**	0.014	**0.041**
0.45	122.91	**<0.001**	0.182	0.070	0.228	0.064	0.241	0.076	0.046	**<0.001**	0.059	**<0.001**	0.013	**0.041**
0.5	123.17	**<0.001**	0.163	0.066	0.206	0.060	0.219	0.071	0.043	**<0.001**	0.055	**<0.001**	0.013	**0.036**
*m = 5*														
0.1	15.41	**<0.001**	0.365	0.046	0.372	0.045	0.352	0.054	0.007	**0.009**	-0.013	**<0.001**	-0.020	**<0.001**
0.15	119.49	**<0.001**	0.364	0.076	0.415	0.058	0.410	0.071	0.051	**<0.001**	0.046	**<0.001**	-0.005	0.634
0.2	126.02	**<0.001**	0.326	0.088	0.386	0.076	0.393	0.093	0.060	**<0.001**	0.066	**<0.001**	0.006	0.604
0.25	124.00	**<0.001**	0.286	0.087	0.345	0.078	0.357	0.096	0.059	**<0.001**	0.070	**<0.001**	0.012	0.192
0.3	122.77	**<0.001**	0.252	0.083	0.307	0.075	0.320	0.093	0.055	**<0.001**	0.068	**<0.001**	0.013	0.097
0.35	122.05	**<0.001**	0.223	0.077	0.274	0.071	0.287	0.086	0.051	**<0.001**	0.064	**<0.001**	0.014	0.061
0.4	122.10	**<0.001**	0.198	0.072	0.246	0.066	0.259	0.080	0.047	**<0.001**	0.060	**<0.001**	0.013	**0.045**
0.45	121.42	**<0.001**	0.178	0.067	0.221	0.062	0.234	0.074	0.044	**<0.001**	0.056	**<0.001**	0.013	**0.036**
0.5	121.54	**<0.001**	0.160	0.062	0.200	0.058	0.213	0.069	0.041	**<0.001**	0.053	**<0.001**	0.012	**0.029**

*F* F-statistic, *SD* standard deviation, *MD* mean difference

Bold values indicate significant differences

For *N* = 600 (i.e. 30 s), in the AP direction, similar trends to those for longer data lengths (*N* = 1200) were observed. Namely, older adults showed generally higher ApEn mean values than young adults, with a decreased consistency in trend (3 trend flips for *N* = 600 versus 1 trend flip for *N* = 1200) (Additional file 1: Figure S3). These differences were statistically significant (*p* < 0.05) for all but one combination of *m* and *r* (Additional file 2: Table S3). In the ML direction, Fallers showed generally lower ApEn mean values than Young adults, in partial agreement with the results obtained for *N* = 1200 (Additional file 1: Figure S4). The dissimilarities observed were that, in contrast to the trend observed for *N* = 1200, the trend observed for *N* = 600 was not consistent for all combinations of *m* and *r* (i.e. some flips appeared for shorter data length) and was found to be statistically significant for some combinations of *m* and *r* (Additional file 2: Table S4). As for the differences between Non-Fallers and Young adults, no consistent trend was observed, in agreement with the results for a data length of *N* = 1200.

##### Older adults, Non-Faller versus Fallers

For *N* = 1200 (i.e. 60 s) in the AP direction, Fallers showed generally higher ApEn mean values than Non-Fallers (Fig. 2). Some exceptions to this trend were found: ApEn(*m* = 4, *r* = 0.1) and ApEn(*m* = 5, *r* = {0.1, 0.15}). However, statistical testing revealed significant differences only for specific parameter combinations (Table 2). In the ML direction, Fallers exhibited lower ApEn mean values than Non-Fallers for all combinations of *m* and *r* (Additional file 1: Figure S2). However, statistical testing revealed that only two combinations of *m* and *r* produced significant differences between groups (Additional file 2: Table S2).

For *N* = 600 (i.e. 30 s) in the AP direction, the relative consistency of ApEn and its ability to discriminate between Non-Fallers and Fallers were challenged. Firstly, more trend flips were observed for shorter time-series (*N* = 600) than for longer ones (*N* = 1200) (Additional file 1: Figure S3). In addition, statistical significance was only observed for combinations of *m* and *r* producing trend flips, thus casting doubt on its legitimacy (Additional file 2: Table S3). In the ML direction, similar trends in group differences were observed for shorter data lengths (*N* = 600) compared to longer data length (*N* = 1200). Namely, Fallers showed generally lower ApEn mean values than Non-Fallers, with a slightly less consistent trend (1 trend flips for *N* = 600 versus any “flip” for *N* = 1200) (Additional file 1: Figure S4). Moreover, in agreement with results for *N* = 1200, only specific combinations of *m* and *r* produced statistically significant trends (Additional file 2: Table S4).

#### Sample entropy

A significant four-way interaction between group, *m*, *r* and *N* was found [Anterior-Posterior: F(4.82, 4571 = 6.71, *p* < 0.001, partial η^2^ = 0.007; Medial-Lateral: F(6.7, 6354) = 2.18, *p* = 0.035, partial η^2^ = 0.002]. This indicated that the SampEn values were different between at least two groups for one or more combinations of *m*, *r* and *N*. Once again, the reported *p*-values are the corrected ones using the Greenhouse-Geisser procedure, given that the compound symmetry assumption was violated (Mauchly’s test with a *p* < 0.001 for both anterior-posterior and medial-lateral COP time-series).

##### Young versus Older adults (Non-Fallers and Fallers)

**Fig. 3:**
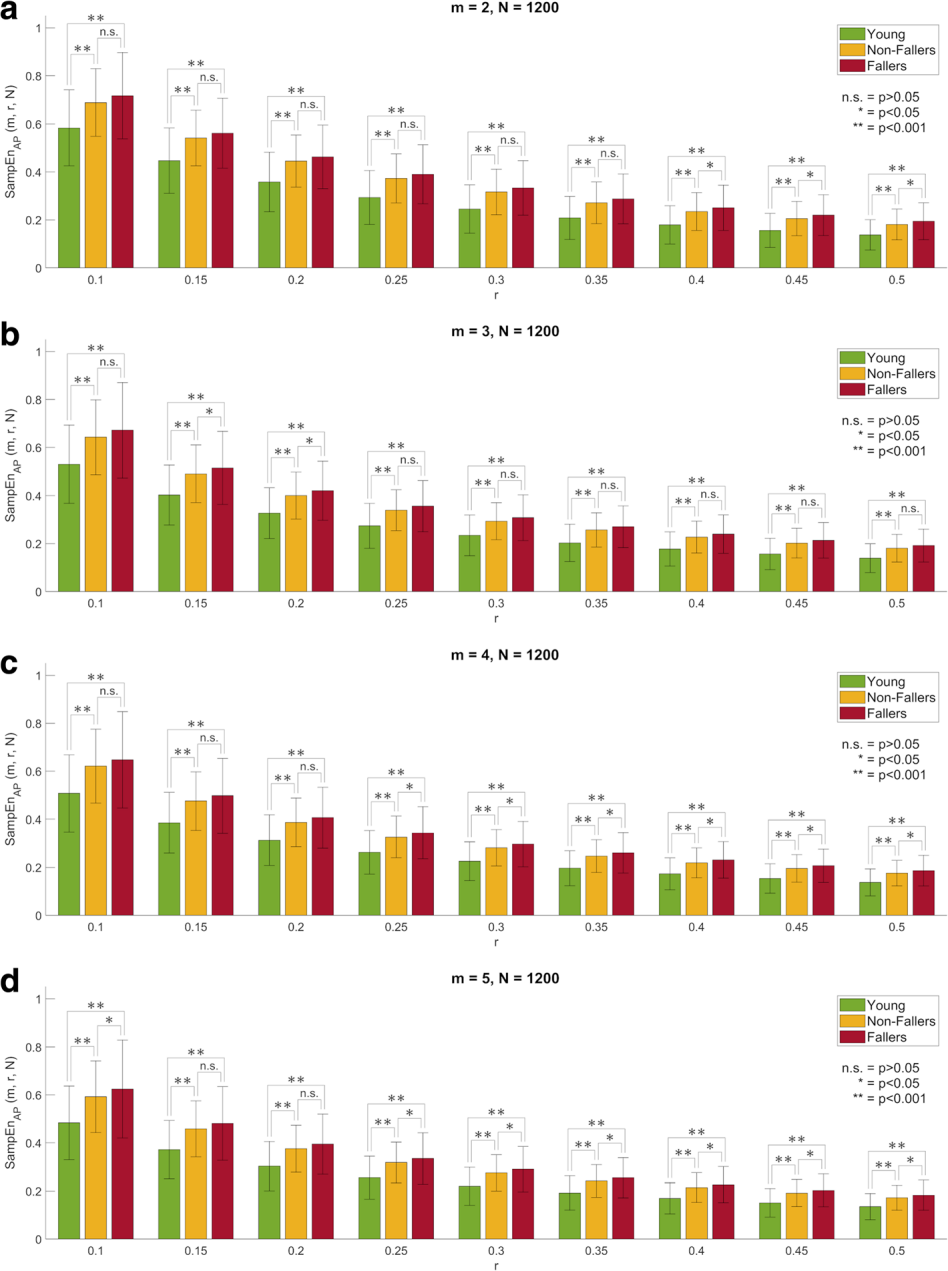
Sample entropy (SampEn) mean value (bars) and standard deviation (error lines) by group as a function of *r* for *m* = {2, 3, 4, 5} (from top to bottom) and *N* = 1200 (i.e. 60 s) for the anterior-posterior (AP) component of the centre of pressure displacement during quiet standing: **a**) *m* = 2, **b**) *m* = 3, **c**) *m* = 4, and **d**) *m* = 5

For *N* = 1200 (i.e. 60 s) in the AP direction, Fallers and Non-Fallers showed higher SampEn mean values than Young adults for all combinations of *m* and *r* (Fig. 3). Those differences were found statistically significant with a *p* < 0.001 (Table 3). In the ML direction, Non-Fallers had higher SampEn mean values than Young adults for all combinations of *m* and *r* (Additional file 1: Figure S5). In contrast, Fallers had generally lower values compared to Young adults. However, all those differences between Young and Non-Fallers/Fallers were found not statistically significant (Additional file 2: Table S5).

**Table 3:** Sample entropy in the anterior-posterior direction as a function of *r* and *m* for a data length of *N*=1200 (i.e. 60-seconds)

	One-way ANOVA		Descriptive statistics by group						Post-hoc					
			Young (Y)		Non-Fallers (NF)		Fallers (F)		NF - Y		F - Y		F - NF	
*r*	F	*p*-value	Mean	SD	Mean	SD	Mean	SD	MD	*p*-value	MD	*p*-value	MD	*p*-value
*m = 2*														
0.1	128.20	**<0.001**	0.583	0.158	0.689	0.140	0.718	0.179	0.106	**<0.001**	0.134	**<0.001**	0.028	0.051
0.15	138.04	**<0.001**	0.447	0.136	0.542	0.116	0.562	0.146	0.094	**<0.001**	0.115	**<0.001**	0.020	0.125
0.2	139.12	**<0.001**	0.358	0.124	0.445	0.109	0.463	0.133	0.087	**<0.001**	0.105	**<0.001**	0.018	0.149
0.25	137.79	**<0.001**	0.294	0.113	0.373	0.102	0.390	0.123	0.080	**<0.001**	0.097	**<0.001**	0.017	0.116
0.3	134.96	**<0.001**	0.245	0.101	0.316	0.095	0.333	0.114	0.071	**<0.001**	0.088	**<0.001**	0.017	0.082
0.35	132.15	**<0.001**	0.208	0.090	0.271	0.087	0.288	0.104	0.063	**<0.001**	0.079	**<0.001**	0.016	0.058
0.4	129.41	**<0.001**	0.179	0.080	0.235	0.079	0.250	0.095	0.056	**<0.001**	0.071	**<0.001**	0.016	**0.040**
0.45	126.97	**<0.001**	0.156	0.071	0.205	0.071	0.220	0.085	0.049	**<0.001**	0.064	**<0.001**	0.015	**0.030**
0.5	125.08	**<0.001**	0.137	0.063	0.181	0.064	0.194	0.077	0.044	**<0.001**	0.057	**<0.001**	0.013	**0.025**
*m = 3*														
0.1	126.15	**<0.001**	0.531	0.163	0.643	0.156	0.672	0.199	0.112	**<0.001**	0.141	**<0.001**	0.029	0.064
0.15	132.96	**<0.001**	0.403	0.125	0.491	0.120	0.515	0.152	0.088	**<0.001**	0.113	**<0.001**	0.025	**0.037**
0.2	133.72	**<0.001**	0.327	0.106	0.401	0.098	0.421	0.124	0.074	**<0.001**	0.094	**<0.001**	0.020	**0.041**
0.25	135.48	**<0.001**	0.274	0.094	0.339	0.085	0.356	0.107	0.065	**<0.001**	0.082	**<0.001**	0.017	0.051
0.3	134.96	**<0.001**	0.234	0.085	0.294	0.077	0.308	0.096	0.059	**<0.001**	0.074	**<0.001**	0.015	0.069
0.35	134.70	**<0.001**	0.203	0.078	0.257	0.071	0.270	0.087	0.054	**<0.001**	0.067	**<0.001**	0.013	0.073
0.4	133.25	**<0.001**	0.178	0.071	0.227	0.066	0.240	0.080	0.050	**<0.001**	0.062	**<0.001**	0.012	0.066
0.45	131.57	**<0.001**	0.157	0.066	0.202	0.062	0.214	0.074	0.045	**<0.001**	0.057	**<0.001**	0.012	0.061
0.5	129.87	**<0.001**	0.139	0.060	0.181	0.058	0.192	0.069	0.042	**<0.001**	0.053	**<0.001**	0.011	0.053
*m = 4*														
0.1	128.60	**<0.001**	0.508	0.161	0.621	0.154	0.647	0.201	0.113	**<0.001**	0.140	**<0.001**	0.026	0.102
0.15	133.16	**<0.001**	0.386	0.126	0.476	0.122	0.498	0.155	0.090	**<0.001**	0.112	**<0.001**	0.022	0.072
0.2	132.77	**<0.001**	0.313	0.105	0.387	0.101	0.407	0.126	0.074	**<0.001**	0.094	**<0.001**	0.019	0.051
0.25	133.28	**<0.001**	0.263	0.091	0.326	0.086	0.344	0.108	0.064	**<0.001**	0.081	**<0.001**	0.018	**0.039**
0.3	132.67	**<0.001**	0.226	0.081	0.282	0.076	0.297	0.095	0.056	**<0.001**	0.072	**<0.001**	0.015	**0.042**
0.35	132.30	**<0.001**	0.196	0.073	0.247	0.068	0.261	0.084	0.051	**<0.001**	0.064	**<0.001**	0.014	**0.046**
0.4	131.41	**<0.001**	0.173	0.067	0.219	0.062	0.231	0.076	0.046	**<0.001**	0.058	**<0.001**	0.012	**0.049**
0.45	130.20	**<0.001**	0.154	0.061	0.196	0.057	0.207	0.070	0.042	**<0.001**	0.053	**<0.001**	0.011	**0.047**
0.5	129.18	**<0.001**	0.137	0.056	0.176	0.053	0.186	0.064	0.039	**<0.001**	0.049	**<0.001**	0.011	**0.045**
*m = 5*														
0.1	130.78	**<0.001**	0.484	0.153	0.592	0.150	0.625	0.204	0.109	**<0.001**	0.141	**<0.001**	0.032	**0.027**
0.15	133.99	**<0.001**	0.371	0.122	0.458	0.117	0.481	0.154	0.087	**<0.001**	0.110	**<0.001**	0.023	0.054
0.2	133.48	**<0.001**	0.303	0.103	0.376	0.098	0.395	0.125	0.073	**<0.001**	0.092	**<0.001**	0.019	0.053
0.25	133.36	**<0.001**	0.255	0.089	0.318	0.085	0.335	0.107	0.063	**<0.001**	0.080	**<0.001**	0.017	**0.045**
0.3	132.80	**<0.001**	0.220	0.079	0.275	0.075	0.290	0.094	0.055	**<0.001**	0.071	**<0.001**	0.015	**0.040**
0.35	131.65	**<0.001**	0.192	0.071	0.241	0.068	0.255	0.084	0.050	**<0.001**	0.063	**<0.001**	0.014	**0.043**
0.4	130.43	**<0.001**	0.169	0.065	0.214	0.062	0.226	0.075	0.045	**<0.001**	0.057	**<0.001**	0.012	**0.046**
0.45	128.72	**<0.001**	0.151	0.059	0.191	0.056	0.203	0.068	0.041	**<0.001**	0.052	**<0.001**	0.011	**0.042**
0.5	127.30	**<0.001**	0.135	0.055	0.172	0.052	0.183	0.062	0.037	**<0.001**	0.048	**<0.001**	0.010	**0.041**

*F* F-statistic, *SD* standard deviation, *MD* mean difference

Bold values indicate significant differences

For *N* = 600 (i.e. 30 s) in the AP direction, the relative trend consistency of SampEn and its ability to discriminate between Young adults and older adults (both Fallers and Non-Fallers) were preserved. Namely, Non-Fallers and Fallers showed higher SampEn mean values than Young adults for all combinations of *m* and r **(**Additional file 1: Figure S6). Those differences remained statistically significant with *p*-values < 0.001 (Additional file 2: Table S6). In the ML direction, some combinations of *m* and *r* produced statistically significant differences between Young adults and Fallers (Additional file 2: Table S7), an unexpected result considering that no significant differences between groups were observed for longer time-series (*N* = 1200). More specifically, Fallers showed lower SampEn mean values than Young adults (Additional file 1: Figure S7). On the other hand, the relative trend consistency in differences between Young adults and Non-Fallers was challenged, corrupting the consistent trend observed for longer time-series (*N* = 1200) for which Non-Fallers showed higher SampEn values than Young adults for all combinations of *m* and *r*.

##### Older adults, Non-Faller versus Fallers

For *N* = 1200 (i.e. 60 s) in the AP direction, Fallers exhibited higher SampEn mean values than Non-Fallers for all combinations of *m* and *r* (Fig. 3). However, statistical testing revealed significant differences only for specific parameter combinations (Table 3). In the ML direction, Fallers exhibited lower SampEn mean values than Non-Fallers for all combinations of *m* and *r* (Additional file 1: Figure S5). No significant differences were found between Non-Fallers and Fallers (Additional file 2: Table S5).

For *N* = 600 (i.e. 30 s) in the AP direction, the ability of SampEn to discriminate between Non-Fallers and Fallers was challenged. Namely, no statistically significant differences between Non-Fallers and Fallers were observed (Additional file 2: Table S6), even if a consistent decrease was preserved (Additional file 1: Figure S6). In the ML direction, two combinations of *m* and *r* produced statistically significant differences between Non-Fallers and Fallers (Additional file 2: Table S7), with Fallers showing lower SampEn mean values than Non-Fallers (Additional file 1: Figure S7). These results differ from the results obtained with longer COP time-series (*N* = 1200), where no significant differences were observed.

#### Linear measures

Both Fallers and Non-Fallers exhibited higher mean values than Young adults for all linear measures of COP displacement. These differences were found statistically significant with a *p* < 0.001. Moreover, Fallers exhibited higher mean values than Non-Fallers for all linear measures. However, those differences did not reach statistical significance (Table 4).

**Table 4:** Linear measures of centre of pressure displacement by group

	Descriptive statistics by group						Post hoc					
	Young (Y)		Non-Fallers (NF)		Fallers (F)		NF-Y		F-Y		F-NF	
Variable	Mean	SD	Mean	SD	Mean	SD	MD	*p*-value	MD	*p*-value	MD	*p*-value
Total displacement (cm)	110.479	71.097	152.342	96.961	162.718	102.772	41.863	**<0.001**	52.239	**<0.001**	10.376	0.266
Standard deviation, AP (cm)	0.730	0.347	0.833	0.434	0.866	0.446	0.103	**<0.001**	0.136	**<0.001**	0.033	0.519
Standard deviation, ML (cm)	0.511	0.279	0.639	0.401	0.683	0.440	0.128	**<0.001**	0.172	**<0.001**	0.044	0.251
Amplitude, AP (cm)	4.017	2.006	4.810	2.646	5.103	2.684	0.793	**<0.001**	1.086	**<0.001**	0.293	0.247
Amplitude, ML (cm)	2.930	1.669	3.663	2.357	3.889	2.575	0.733	**<0.001**	0.959	**<0.001**	0.226	0.337
Total Mean Velocity, (cm/s)	1.841	1.185	2.539	1.616	2.712	1.713	0.698	**<0.001**	0.871	**<0.001**	0.173	0.266
Mean Velocity, AP (cm)	1.313	0.862	1.928	1.212	2.118	1.343	0.615	**<0.001**	0.805	**<0.001**	0.190	0.058
Mean Velocity, ML (cm)	1.013	0.668	1.261	0.864	1.273	0.846	0.248	**<0.001**	0.260	**<0.001**	0.012	0.981
Area (cm2)	8.270	7.481	12.71	13.015	14.25	14.326	4.440	**<0.001**	5.980	**<0.001**	1.540	0.155

*SD* standard deviation, *MD* mean difference, *AP* anterior-posterior, *ML* medial-lateral

Bold values indicate significant differences

### Behaviour of SampEn under the different testing conditions

**Fig. 4:**
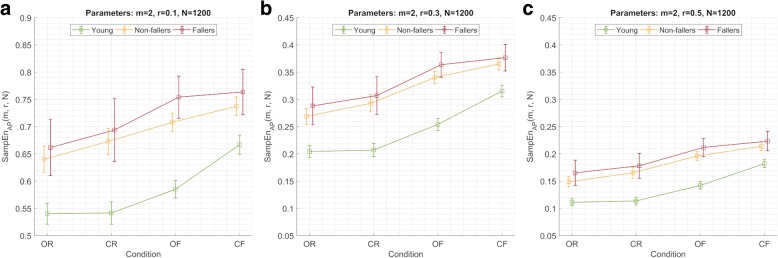
Sample entropy (SampEn) mean value (marker) and 95% confidence interval (error lines) by group and testing condition for selected parameter combinations: **a**) SampEn_AP_(*m* = 2, *r* = 0.1, *N* = 1200), **b**) SampEn_AP_(*m* = 2, *r* = 0.3, *N* = 1200), and **c**) SampEn_AP_(*m* = 2, *r* = 0.5, *N* = 1200). Conditions (vision-surface): *OR* open-rigid, *CR* closed-rigid, *OF* open-foam, *CF* closed-foam

For any given parameter combination, the mean SampEn value by group increased across the four testing conditions (vision-surface): open-rigid (OR) < closed-rigid (CR) < open-foam (OF) < closed-foam (CF). Older adults showed higher mean SampEn values than young adults across all testing conditions, with Fallers consistently exhibiting higher mean values than Non-Fallers. The differences between older and young adults were found to be significant for all parameter combinations across testing conditions (Additional file 2: Tables S8–S11). However, significant differences between Non-Fallers and Fallers were only found under the OF condition for two parameter combinations (Additional file 2: Table S10). To illustrate these findings, Fig. 4 shows SampEn mean value and 95% confidence interval by group and testing condition for three selected parameter combinations, one of which produced significant differences between Fallers and Non-fallers (*m* = 2, *r* = 0.1, *N* = 1200).

## Discussion

The use of Approximate Entropy (ApEn) and Sample Entropy (SampEn) to characterise the regularity of COP trajectories is still in practice. While previous studies have achieved promising results regarding the use of those entropy measures to discriminate between experimental groups and/or testing conditions, the adequate selection of input parameter values for the analysis of COP time-series has been scarcely investigated. This study aimed (1) to examine the effect of changing the values of parameters *m*, *r* and *N* on ApEn and SampEn values in COP time-series, and (2) to determine the ability of ApEn and SampEn to discriminate between experimental groups. It was expected that ApEn and SampEn values would change significantly as a function of *m*, *r* and *N*, yet that SampEn would maintain a consistent behaviour across different parameter value combinations (e.g. young adults showing consistently either higher or lower entropy values than older adults) [27]. Moreover, it was expected that significant differences in entropy values between young and older adults would be observed and that some parameter value combinations would potentially reveal significant differences between non-fallers and fallers.

Firstly, our results confirm that the ApEn and SampEn algorithms are very sensitive to input parameter choice. Consequently, researchers and clinicians should be cautious when comparing studies using different parameters, even in similar populations and testing conditions: a direct comparison of entropy values (e.g. mean and range) should be completely avoided. However, our analyses allow observing the behaviour of ApEn and SampEn mean values over a wide range of input parameters, which might be useful for other studies. Namely, for a chosen *m*, both ApEn and SampEn tended to decrease as *r* increased, except in the case of ApEn for low values of *r* in combination with high values of *m*. The decreasing trend showed steeper slopes for lower values of *m*. Similarly, for a chosen *r*, ApEn and SampEn tended to decrease as *m* increased (Fig. 1). In other words, COP time-series exhibited more regularity (i.e., lower entropy values) for higher similarity tolerances and higher subseries lengths. The increase in regularity for higher values of *r* seems to be an expected result, as it is a reasonable assumption that a higher number of subseries will meet the similarity criterion for a more relaxed tolerance. The increase in regularity for higher values of *m* suggests that patterns in COP time-series are observed at larger time-scales rather than at smaller time-scales (e.g. in our study, *m* = 5 would correspond to a 0.25 to 0.3-s pattern and *m* = 2 to a 0.1 to 0.15-s pattern). This could be presumably linked to the well-known fact that for the quiet standing posture the main components of the COP signal are below 10 Hz [6]. As for the effects of data length, our results confirmed that ApEn is more dependent on this parameter than SampEn [26]. This claim is supported by the lower ApEn values observed for shorter time-series (*N* = 600) than for longer time-series (*N* = 1200). This situation is particularly evident for higher *m* values and lower *r* values. For instance, refer to Fig. 1 to compare ApEn_AP_(*m* = 5, *r* = 0.1) for *N* = 600 (top left pane) to *N* = 1200 (top right pane); then compare SampEn_AP_ (*m* = 5, *r* = 0.1) for *N* = 600 (bottom left pane) to *N* = 1200 (bottom right pane). Whereas a difference in the ApEn value between longer and shorter time-series is evident, the difference in the SampEn value is barely noticeable. These initial findings already tipped the scales in favour of SampEn when dealing with COP time-series, in line with previous studies that had suggested their use for the analysis of inter-beat interval, gait and brain activity time-series [26–28]. Otherwise, they allowed us to narrow down the number of potentially useful input parameter combinations in case of using ApEn, discarding combinations of *m* = {4,5} and *r* = {0.1, 0.15, 0.2}.

Secondly, our results highlighted issues with relative consistency in COP time-series for ApEn, as observed by the change in direction of differences between groups (known as “flips” or “crossovers”) for some combinations of *m* and *r*. For instance, in the AP direction older adults with falls in the last 12 months (Fallers) showed generally higher ApEn mean values than young adults, but the opposite trend was observed for ApEn(*m* = 5, *r* = 0.1). This issue was still more evident when comparing older adults with and without falls in the last 12 months (i.e. Non-fallers and Fallers, respectively) as more combinations of *m* and *r* produce crossovers. Moreover, the issue with relative consistency was accentuated for shorter time-series (*N* = 600). Importantly, these issues were observed for higher values of *m* and lower values of *r*, which once again suggest that these values are not an optimal choice for COP time-series analysis based on ApEn. In contrast, SampEn showed relative consistency, as no crossovers between groups were observed. This feature has been highlighted as one of the advantages of SampEn over ApEn for other types of biological data analysis as well [26–28]. This is an additional reason why researchers and clinicians should favour SampEn over ApEn for analysing COP time-series.

Additionally, our results suggested that ApEn and SampEn are more sensitive than traditional COP displacement measures to differences between groups: ApEn and SampEn were able to discriminate between older adults with and without falls in the last 12 months, whereas linear measures were not. In other words, while Non-fallers and Fallers exhibited commensurable COP displacements in terms of magnitude (i.e. total length, amplitude and area), variability (i.e. standard deviation) and velocity, they manifested differences in COP time-series structure (more specifically, in regularity). Nevertheless, our findings also revealed that the selection of input parameters in the computation of ApEn and SampEn is critical in the identification of significant differences between groups. Certainly, ApEn and SampEn were able to discriminate with ease between two highly heterogeneous groups, i.e. young and older adults, for a wide range of *m*, *r* and *N* values. However, only a subset of combinations revealed significant differences between more homogeneous groups; i.e., older adults with and without falls in the last 12 months. Those differences between groups were mainly observed for COP time-series in the anterior-posterior direction with longer length (*N* = 1200, equivalent to a 60-s duration). Moreover, SampEn revealed significant differences for a higher number of combinations than ApEn. Therefore, we suggest that researchers and clinicians should aim to collect at least 60 s of posturography data and focus on the analysis of the anterior-posterior component of the COP displacement using SampEn. In those cases where collecting 60 s could prove unfeasible due to the inability of subjects to stand still for longer periods, then they may want to consider alternative nonlinear measures that are less sensitive to data length (a book chapter by Melillo et al. offers a good starting point to explore other nonlinear methods for the analysis of biomedical signals [35]).

Furthermore, a more in-depth analysis of SampEn behaviour under four different testing conditions revealed that, while SampEn is able to discriminate with ease between two highly heterogeneous groups (i.e. young and older adults) for a wide range of testing conditions, some specific conditions might boost its sensitivity to differences between more homogeneous groups (i.e. older adults with and without falls in the last 12 months). Namely, older adults show significantly higher mean values than young adults across all testing conditions. However, significant differences between Non-Fallers and Fallers were only found for one condition; namely, the eyes open-foam surface condition (OF). Certainly, this was the case only for two parameter combinations. However, this fact might be explained by the imbalance in the dataset: there were 85 (53.5%) young adults, 56 (35.2%) non-fallers and only 18 (11.3%) fallers. These numbers have an important impact on inferential statistics: with a particularly low number of subjects in the Fallers group, the 95% confidence interval for the mean (95% CI) of the group is expected to be wide, thus overlapping with the 95% CI of the Non-fallers group. This situation is illustrated in Fig. 4, where SampEn mean values and 95% CI by group and condition are shown for three selected parameter combinations. It can be observed that the 95% CIs for the Non-fallers and Fallers groups in the OF condition only partially overlap, suggesting that given a higher number of subjects in the Fallers group its 95% CI would shrink, potentially producing non-overlapping 95% CIs between those two groups. In contrast, the Non-Fallers and Fallers 95% CI for other testing conditions are totally or almost totally overlapping, suggesting that they would remain so even if the size of the former group was higher. Similar results were observed across all values of *m* considered in the present study, thus its choice seems to play a minor role in this specific aspect of analysis. However, our results suggest that the choice of *r* is critical, as higher values of *r* (e.g. *r* = 0.5) seem to distort the potentially distinctive ‘profile line’ that each group shows for lower values (e.g. *r* = 0.1) when SampEn mean values are plotted across testing conditions. This observation allow us to further narrow down the options of potentially useful values of *r* to somewhere in the middle of the range (e.g. *r* = {0.25, 0.3, 0.35}).

From the clinical perspective, our findings provide researchers and practitioners with interesting insights. The first one has to do with the direction of the difference in entropy values between the experimental groups in our study (i.e. young adults and older adults with and without recent falls). In the anterior-posterior direction, older adults (both fallers and non-fallers) exhibited significantly higher entropy values than young adults for most combinations of *m*, *r* and *N*. Moreover, Fallers exhibited generally higher SampEn values than Non-Fallers (although that difference was significant only for some combinations of input parameters). Therefore, our findings conflict with the traditional interpretation of entropy values, which suggest that older adults should exhibit generally lower entropy values as a consequence of the loss of physiological complexity due to ageing and ill-health [36]. This conflict is solved by bearing in mind that entropy cannot be directly linked to complexity: a smaller entropy value does not mean less complex, it only indicates more regularity based on one particular timescale [25, 26]. Therefore, if COP entropy values observed in healthy young adults are to be taken as a reference, then the higher values found in older adults (especially in Fallers) may be indicative of posture control mechanisms that are too random to properly command balance. In other words, the irregularity observed in older adults might be associated with an unstructured system which becomes less sustainable [17]. As for COP entropy values in the ML direction, the observed results resist a straightforward interpretation, as no significant differences between groups were found. However, the generally lower entropy values observed in Fallers compared to Young adults and Non-Fallers may suggest posture control mechanisms that are too stiff (too regular), which could be problematic when coping with external factors demanding an adaptable balance control. A second insight relates to the sensitivity of entropy measures to differences between groups compared to that of traditional measures. While the traditional measures were only able to discriminate between highly heterogeneous groups (young adults versus older adults), entropies could also discriminate between more homogeneous groups (non-fallers versus fallers). This suggests that Fallers suffer from balance impairments of a different nature to those produced by normal ageing. However, the elucidation of the specific nature of those impairments is beyond the scope of this work. A third insight relates to the conditions that seem to accentuate the differences in balance control mechanisms between our experimental groups. Our findings suggest that neither the least nor the most challenging testing conditions (vision-surface: open-rigid and closed-foam, respectively) enable the discrimination of differences between Non-fallers and Fallers: both groups seem to cope similarly with those conditions. In contrast, a testing condition of intermediate complexity (i.e. open-foam) seems to better reveal those differences.

Finally, we acknowledge that there are more recent developments in the field of nonlinear analysis that could potentially improve the sensitivity when looking for differences between groups. In particular, the development of multiscale entropy (MSE) and multivariate multiscale entropy (MMSE) have offered new perspectives for the analysis of biological time-series [37–40]. A few studies have already applied these approaches to the analysis of COP time-series [13, 20, 22]. Briefly, these approaches rely on the computation of sample entropy values at different time-scales and produce a two-dimensional plot (time-scale versus sample entropy) depicting a profile line for each experimental group/condition. An overall entropy ‘score’ can be computed by adding the entropy values at individual time-scales [20]. While these new approaches represent an interesting tool to explore the level of regularity contained at different time-scales, they cannot avoid the issue of the adequate selection of input parameters. Since MSE and its variations (e.g. MMSE) are based on SampEn, the researchers and clinicians that opt for these newer approaches face essentially the same problem that those who opt for ‘single-scale’ entropy measures when it comes to input parameter selection. Hopefully, the present work will aid them in their choices or at least inspire them to adopt a systematic approach to the identification of the optimal parameters.

## Conclusions

In summary, our findings suggest that SampEn represents a better choice for the analysis of COP time-series given its relative consistency and ability to discriminate between experimental groups. Nevertheless, the selection of input parameter values proved to be critical in the identification of significant differences between groups, in particular when those groups a presumably close to each other (for instance, older adults with and without falls in the last 12 months). Firstly, significant differences were mostly observed in COP time-series in the anterior-posterior direction of 60-s duration (*N* = 1200). Therefore, future studies using these entropy measures should favour longer COP recordings (e.g. ≥ 60 s) over shorter COP recordings (e.g. 30 s), as well as be focus the analyses on anterior-posterior time-series. Additionally, significant differences between groups with a consistent trend were mostly observed for sample entropy. Hence, future studies should favour the use of the latter over approximate entropy. More specifically, when analysing the data regardless of testing condition, significant differences were observed for SampEn(*m* = 2, *r* = {0.4, 0.45, 0.5}) and SampEn(m = {4, 5}, r = {0.25, 0.3, 0.35, 0.4, 0.45, 0.5}). Nevertheless, when analysing the data for specific testing conditions, higher values of *r* (≥4) distorted the seemly distinctive pattern that each group showed when plotting SampEn mean values across testing conditions. All in all, we would suggest researchers and clinicians working on the analysis of COP time-series: 1) to use sample entropy with input parameters *m* = {4,5} and *r* = {0.25, 0.3,0.35}, 2) to focus the analysis on the anterior-posterior component, and 3) to further explore the ‘eyes open-foam surface’ testing condition as a potential booster of differences between groups.

## Additional files

Additional file 1: Supplementary figures. This file contains supplementary Figures S1 to S7. (PDF 1615 kb)

Additional file 2: Supplementary tables. This file contains supplementary Tables S2 to S11. (PDF 2739 kb)

Additional file 3: Dataset. This file contains the approximate entropy and sample entropy values computed from the original COP time-series for the seventy-two combinations of parameter values reported in the manuscript. (CSV 9295 kb)

## Abbreviations

95%CI: 95% confidence interval of the mean
AP: Anterior-posterior
ApEn: Approximate entropy
COP: Centre of pressure
F: older adults with one or more falls in the last 12 months.
MD: Mean difference
ML: Medial-lateral
NF: older adults without falls in the last 12 months
SampEn: Sample entropy
SD: Standard deviation
Y: Young adults
