# Supplementary material for: On the use of approximate entropy and sample entropy with centre of pressure time-series
Source: J Neuroeng Rehabil. 2018 Dec 12;15:116. doi: 10.1186/s12984-018-0465-9 (PMC6291990; doi:10.1186/s12984-018-0465-9)
Supplement: Supplementary file 1 — Supplementary figures. This file contains supplementary Figures S1 to S7. (PDF 1615 kb) [file 12984_2018_465_MOESM1_ESM.pdf]

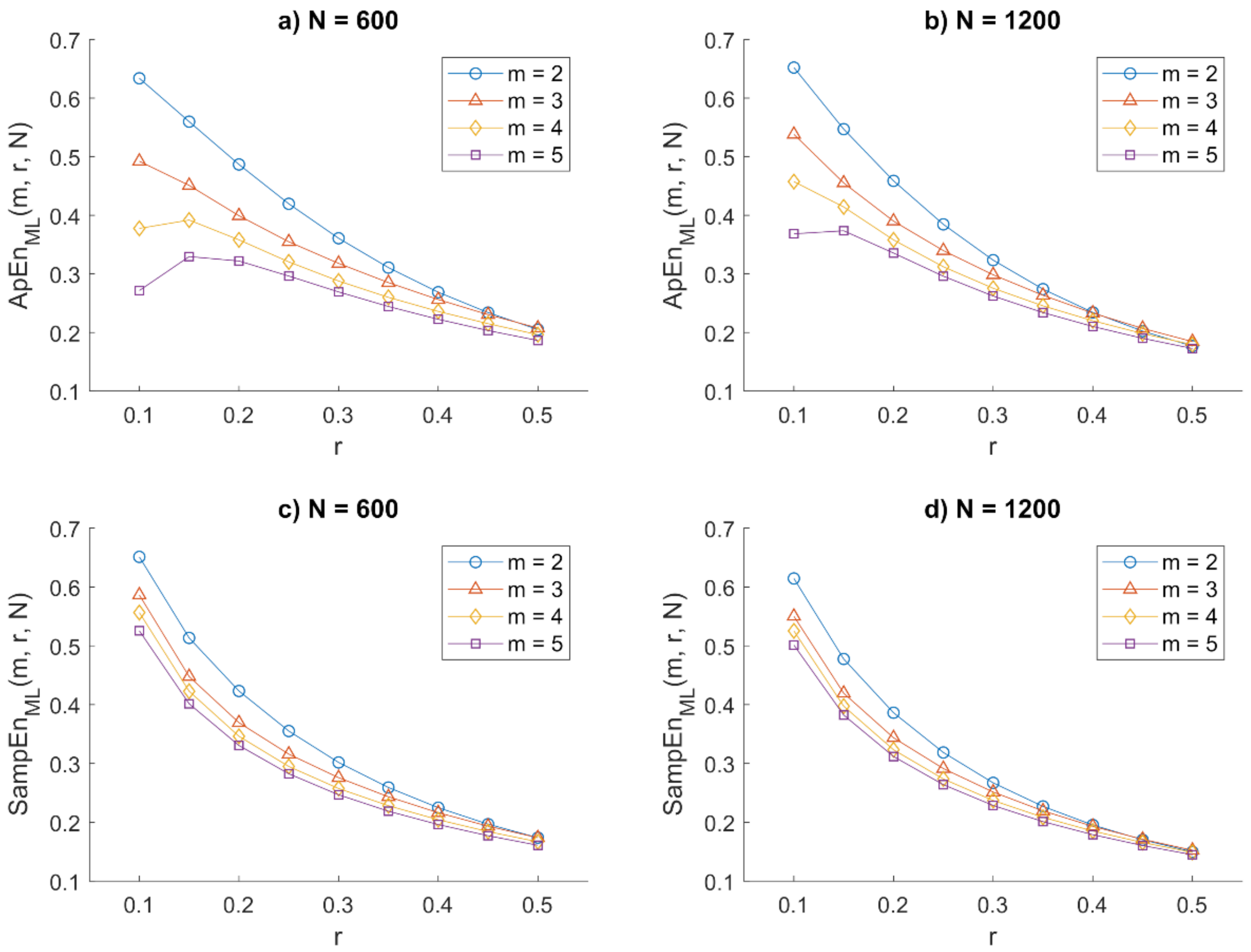

Fig. S1 Approximate entropy (ApEn) and sample entropy (SampEn) as a function of *m*, *r* and *N* for the medial-lateral (ML) component of the centre of pressure displacement during quiet standing.

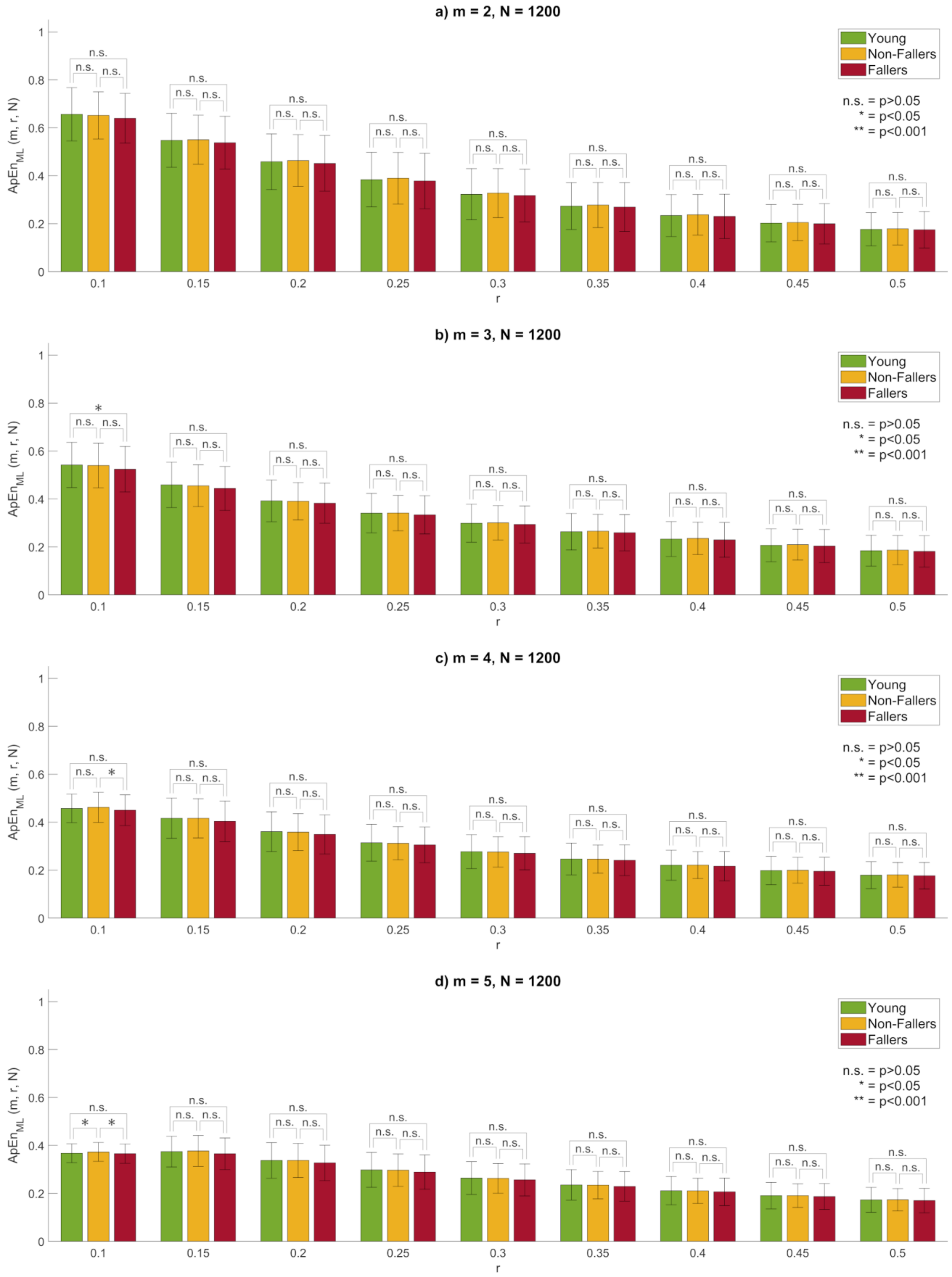

Fig. S2 Approximate entropy (ApEn) mean value (bars) and standard deviation (error lines) by group as a function of r for $m = \{2, 3, 4, 5\}$ (from top to bottom) and $N = 1200$ (i.e. 60 seconds) for the medial-lateral (ML) component of the centre of pressure displacement during quiet standing.

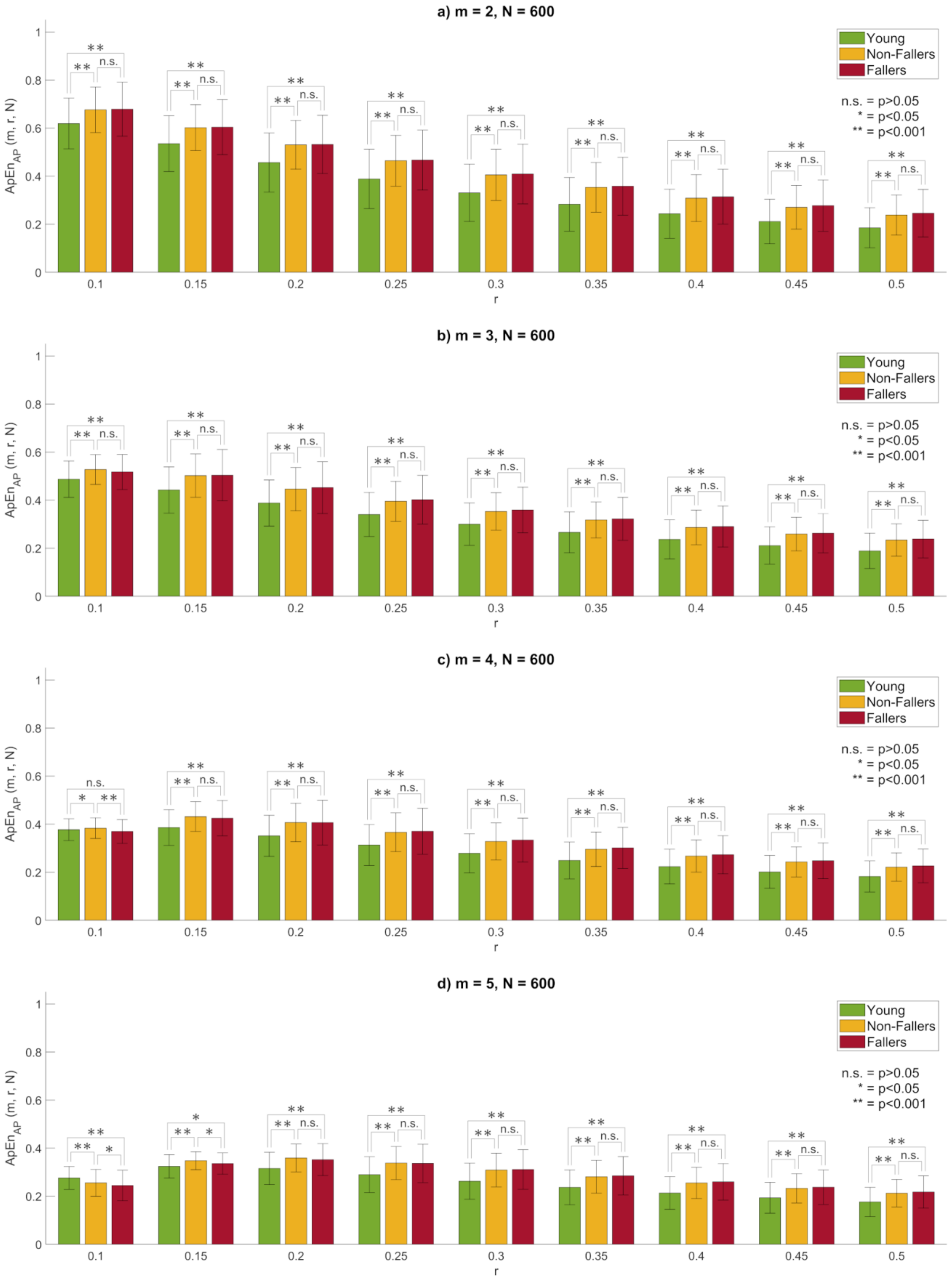

Fig. S3 Approximate entropy (ApEn) mean value (bars) and standard deviation (error lines) by group as a function of r for m = {2, 3, 4, 5} (from top to bottom) and N = 600 (i.e. 30 seconds) for the anterior-posterior (AP) component of the centre of pressure displacement during quiet standing.

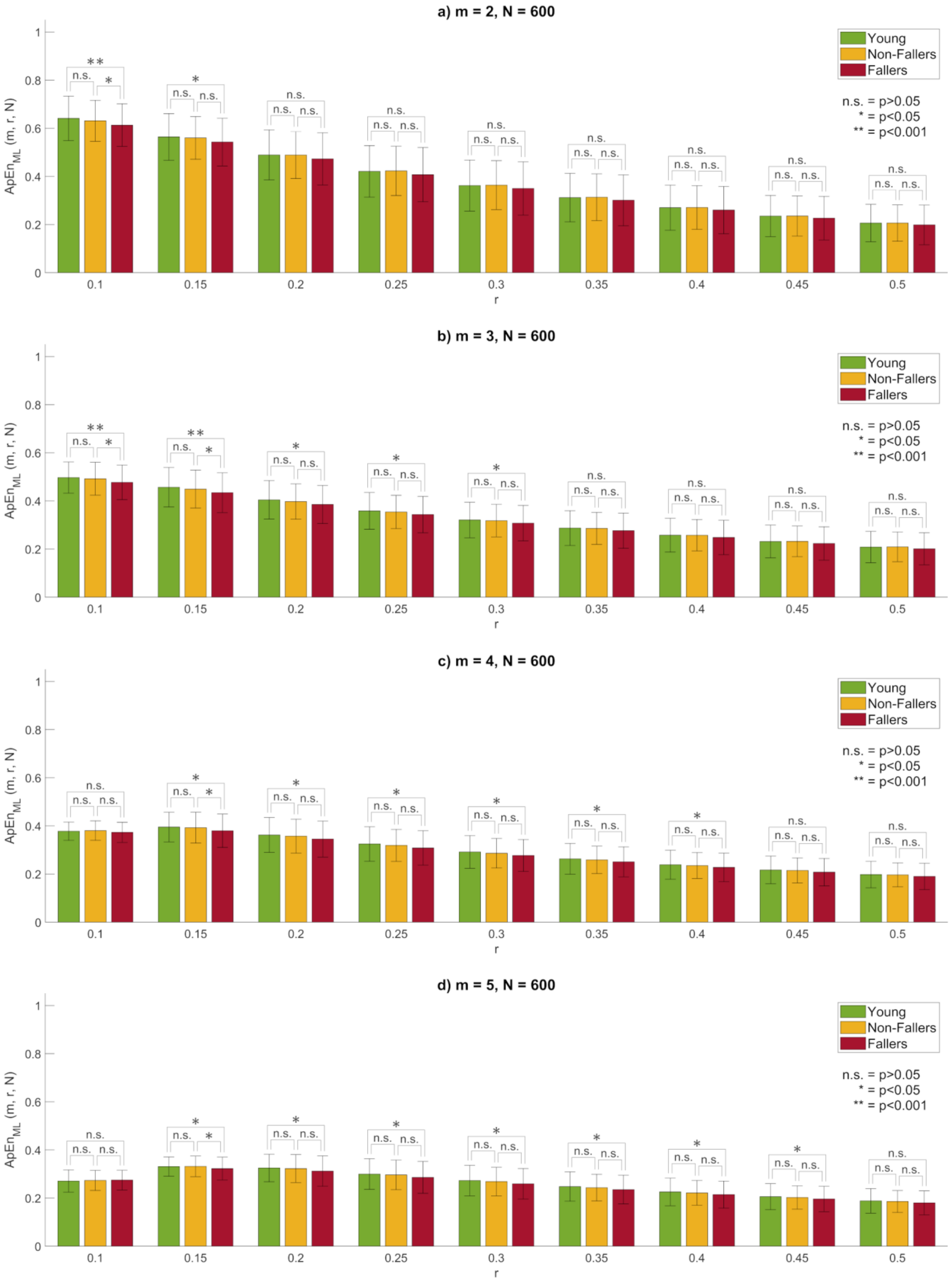

Fig. S4 Approximate entropy (ApEn) mean value (bars) and standard deviation (error lines) by group as a function of *r* for $m = \{2, 3, 4, 5\}$ (from top to bottom) and $N = 600$ (i.e. 30 seconds) for the medial-lateral (ML) component of the centre of pressure displacement during quiet standing.

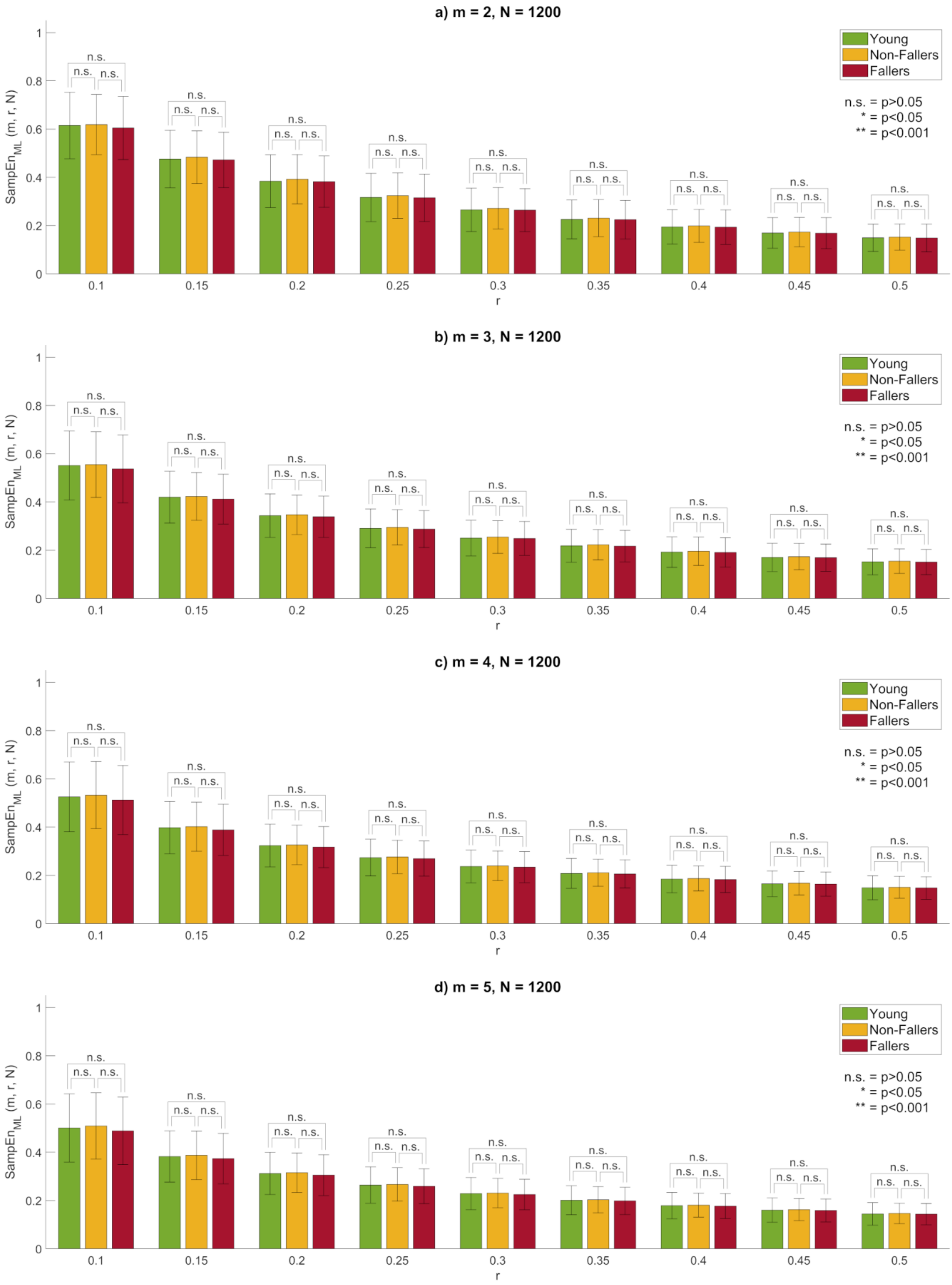

Fig. S5 Sample entropy (SampEn) mean value (bars) and standard deviation (error lines) by group as a function of r for $m = \{2, 3, 4, 5\}$ (from top to bottom) and $N = 1200$ (i.e. 60 seconds) for the medial-lateral (ML) component of the centre of pressure displacement during quiet standing.

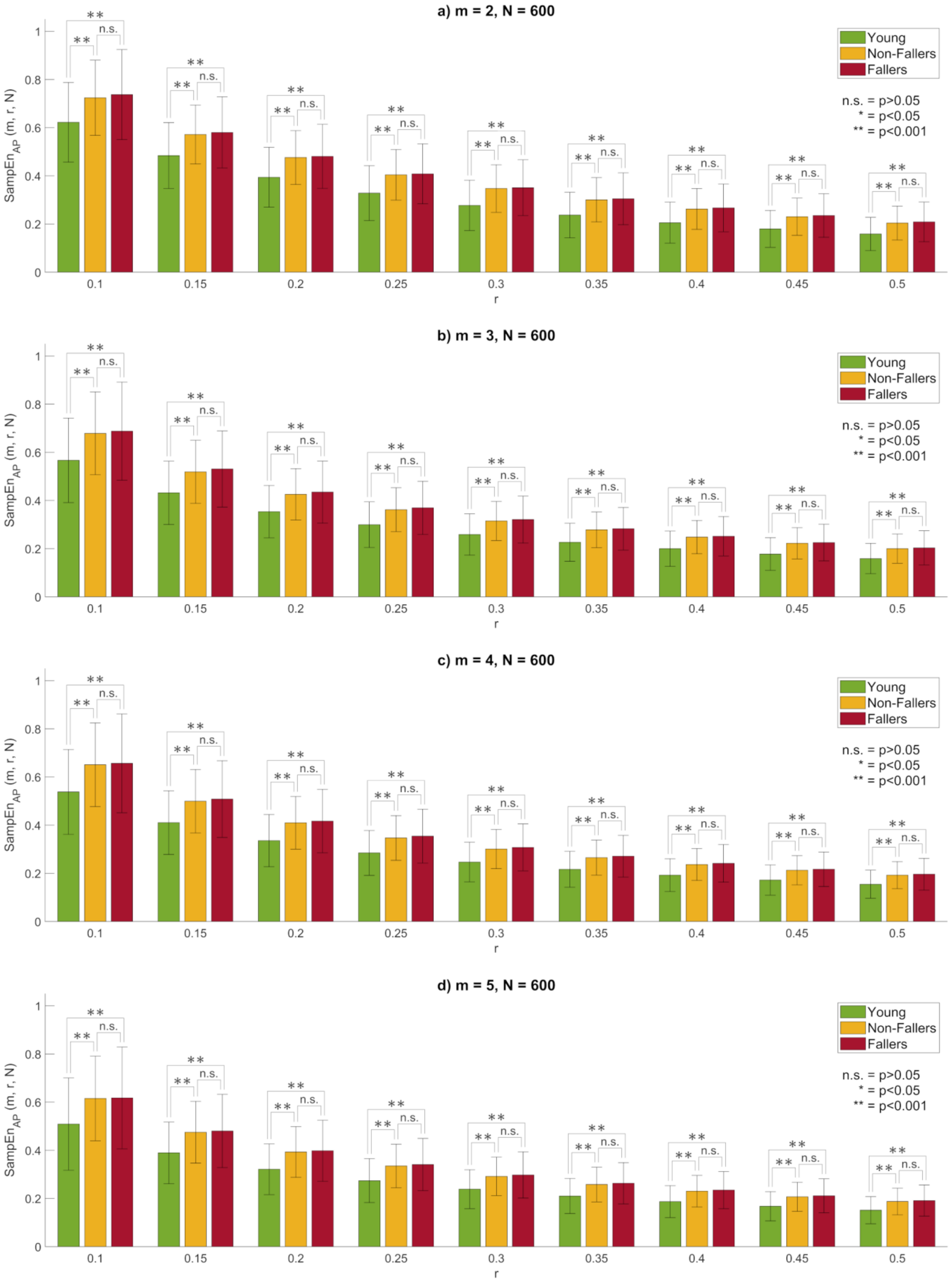

Fig. S6 Sample entropy (SampEn) mean value (bars) and standard deviation (error lines) by group as a function of r for m = {2, 3, 4, 5} (from top to bottom) and N = 600 (i.e. 30 seconds) for the anterior-posterior (AP) component of the centre of pressure displacement during quiet standing.

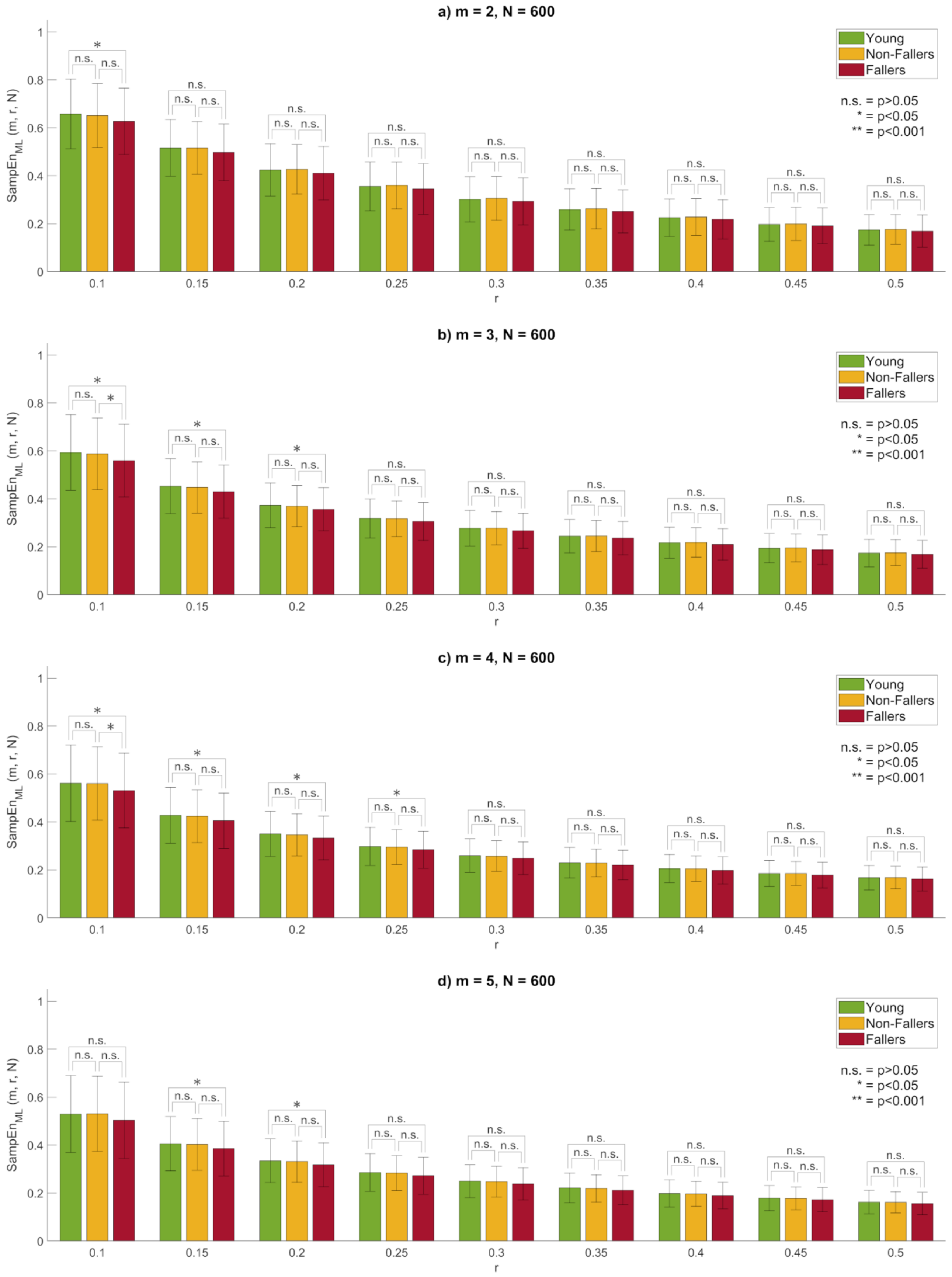

Fig. S7 Sample entropy (SampEn) mean value (bars) and standard deviation (error lines) by group as a function of r for $m = \{2, 3, 4, 5\}$ (from top to bottom) and $N = 600$ (i.e. 30 seconds) for the medial-lateral (ML) component of the centre of pressure displacement during quiet standing.
