# Supplementary material for: On the use of approximate entropy and sample entropy with centre of pressure time-series
Source: J Neuroeng Rehabil. 2018 Dec 12;15:116. doi: 10.1186/s12984-018-0465-9 (PMC6291990; doi:10.1186/s12984-018-0465-9)
Supplement: Supplementary file 2 — Supplementary tables. This file contains supplementary Tables S2 to S11. (PDF 2739 kb) [file 12984_2018_465_MOESM2_ESM.pdf]

Table S2 Approximate entropy in the medial-lateral direction as a function of *r* and *m* for a data length of *N*=1200 (i.e. 60-seconds).

	**One-way ANOVA**		**Descriptive statistics by group**						**Post-hoc**					
			**Young (Y)**		**Non-Fallers (NF)**		**Fallers (F)**		**NF - Y**		**F - Y**		**F - NF**	
r	**F**	**p-value**	**Mean**	**SD**	**Mean**	**SD**	**Mean**	**SD**	**MD**	**p-value**	**MD**	**p-value**	**MD**	**p-value**
m = 2														
0.1	2.21	0.110	0.656	0.111	0.651	0.098	0.639	0.104	-0.005	0.660	-0.017	0.096	-0.012	0.324
0.15	1.05	0.349	0.548	0.113	0.550	0.102	0.538	0.110	0.002	0.898	-0.010	0.442	-0.012	0.317
0.2	0.97	0.380	0.458	0.116	0.463	0.108	0.451	0.116	0.005	0.638	-0.007	0.713	-0.012	0.382
0.25	0.97	0.378	0.384	0.114	0.389	0.108	0.378	0.116	0.006	0.577	-0.006	0.776	-0.011	0.405
0.3	0.87	0.419	0.323	0.107	0.328	0.102	0.318	0.110	0.005	0.611	-0.005	0.798	-0.010	0.447
0.35	0.73	0.481	0.273	0.098	0.278	0.094	0.269	0.102	0.004	0.657	-0.004	0.832	-0.008	0.510
0.4	0.61	0.541	0.234	0.088	0.237	0.085	0.230	0.093	0.003	0.709	-0.004	0.850	-0.007	0.564
0.45	0.49	0.612	0.202	0.078	0.205	0.076	0.199	0.084	0.003	0.742	-0.003	0.895	-0.005	0.643
0.5	0.40	0.673	0.176	0.069	0.179	0.067	0.174	0.076	0.002	0.772	-0.002	0.928	-0.004	0.711
m = 3														
0.1	3.14	**0.043**	0.542	0.094	0.540	0.093	0.524	0.095	-0.002	0.889	-0.018	**0.033**	-0.016	0.090
0.15	2.30	0.100	0.459	0.095	0.455	0.087	0.444	0.092	-0.003	0.738	-0.015	0.082	-0.011	0.256
0.2	1.20	0.303	0.392	0.087	0.390	0.078	0.382	0.084	-0.002	0.916	-0.010	0.269	-0.008	0.437
0.25	0.74	0.477	0.341	0.082	0.341	0.074	0.334	0.080	0	0.999	-0.007	0.476	-0.007	0.488
0.3	0.62	0.536	0.299	0.079	0.300	0.072	0.294	0.077	0.002	0.913	-0.005	0.642	-0.007	0.504
0.35	0.65	0.520	0.263	0.076	0.266	0.070	0.259	0.075	0.002	0.798	-0.004	0.735	-0.007	0.501
0.4	0.68	0.509	0.233	0.073	0.236	0.068	0.229	0.073	0.003	0.735	-0.004	0.784	-0.006	0.509
0.45	0.70	0.498	0.207	0.069	0.209	0.065	0.204	0.069	0.003	0.694	-0.003	0.813	-0.006	0.513
0.5	0.66	0.515	0.184	0.064	0.187	0.061	0.181	0.065	0.003	0.693	-0.003	0.835	-0.005	0.537
m = 4														
0.1	3.26	**0.039**	0.458	0.060	0.462	0.063	0.450	0.064	0.004	0.336	-0.008	0.211	-0.012	**0.033**
0.15	2.34	0.096	0.417	0.084	0.416	0.082	0.403	0.085	0	0.998	-0.013	0.089	-0.013	0.118
0.2	1.82	0.163	0.361	0.083	0.359	0.077	0.349	0.082	-0.002	0.882	-0.012	0.137	-0.010	0.280
0.25	1.31	0.269	0.314	0.077	0.312	0.069	0.305	0.075	-0.002	0.831	-0.009	0.241	-0.007	0.468
0.3	0.87	0.419	0.277	0.071	0.276	0.063	0.270	0.069	-0.001	0.932	-0.007	0.385	-0.006	0.554
0.35	0.61	0.543	0.246	0.066	0.246	0.059	0.241	0.064	0	0.995	-0.005	0.521	-0.005	0.590
0.4	0.47	0.627	0.221	0.063	0.221	0.056	0.217	0.061	0.001	0.983	-0.004	0.658	-0.004	0.612
0.45	0.44	0.645	0.198	0.059	0.200	0.054	0.195	0.058	0.001	0.920	-0.003	0.751	-0.004	0.618
0.5	0.46	0.633	0.179	0.056	0.181	0.051	0.177	0.055	0.001	0.861	-0.003	0.799	-0.004	0.615
m = 5														
0.1	4.98	**0.007**	0.367	0.039	0.373	0.039	0.365	0.040	0.006	**0.013**	-0.002	0.814	-0.007	**0.046**
0.15	2.63	0.072	0.374	0.064	0.377	0.064	0.365	0.066	0.003	0.654	-0.009	0.164	-0.012	0.057
0.2	1.88	0.153	0.337	0.074	0.337	0.071	0.327	0.074	0	1	-0.010	0.146	-0.010	0.173
0.25	1.47	0.230	0.298	0.073	0.297	0.068	0.289	0.072	-0.001	0.949	-0.009	0.202	-0.008	0.320
0.3	1.21	0.300	0.264	0.068	0.263	0.062	0.256	0.067	-0.001	0.902	-0.008	0.266	-0.006	0.446
0.35	0.93	0.396	0.235	0.064	0.234	0.057	0.229	0.061	-0.001	0.926	-0.006	0.362	-0.005	0.535
0.4	0.60	0.547	0.211	0.059	0.211	0.053	0.207	0.057	-0.001	0.975	-0.005	0.516	-0.004	0.631
0.45	0.44	0.645	0.191	0.055	0.191	0.049	0.187	0.054	0	0.999	-0.004	0.630	-0.004	0.676
0.5	0.33	0.717	0.173	0.052	0.173	0.047	0.170	0.051	0	0.991	-0.003	0.738	-0.003	0.707

F F-statistic, *SD* standard deviation, *MD* mean difference
Bold values indicate significant differences

Table S3 Approximate entropy in the anterior-posterior direction as a function of *r* and *m* for a data length of *N*=600 (i.e. 30-seconds).

	**One-way ANOVA**		**Descriptive statistics by group**						**Post-hoc**					
			**Young (Y)**		**Non-Fallers (NF)**		**Fallers (F)**		**NF - Y**		**F - Y**		**F - NF**	
r	**F**	**p-value**	**Mean**	**SD**	**Mean**	**SD**	**Mean**	**SD**	**MD**	**p-value**	**MD**	**p-value**	**MD**	**p-value**
m = 2														
0.1	74.40	**<0.001**	0.619	0.106	0.676	0.095	0.678	0.112	0.057	**<0.001**	0.060	**<0.001**	0.003	0.949
0.15	88.83	**<0.001**	0.535	0.116	0.602	0.096	0.604	0.114	0.066	**<0.001**	0.068	**<0.001**	0.002	0.969
0.2	96.40	**<0.001**	0.457	0.123	0.530	0.101	0.532	0.121	0.073	**<0.001**	0.075	**<0.001**	0.002	0.979
0.25	99.33	**<0.001**	0.388	0.124	0.464	0.106	0.467	0.125	0.076	**<0.001**	0.079	**<0.001**	0.003	0.949
0.3	100.93	**<0.001**	0.331	0.119	0.405	0.107	0.409	0.124	0.075	**<0.001**	0.078	**<0.001**	0.003	0.924
0.35	101.02	**<0.001**	0.283	0.112	0.353	0.103	0.358	0.120	0.071	**<0.001**	0.075	**<0.001**	0.005	0.845
0.4	100.23	**<0.001**	0.243	0.102	0.308	0.098	0.314	0.114	0.065	**<0.001**	0.071	**<0.001**	0.006	0.744
0.45	98.87	**<0.001**	0.211	0.093	0.270	0.091	0.277	0.107	0.059	**<0.001**	0.066	**<0.001**	0.007	0.610
0.5	98.33	**<0.001**	0.185	0.084	0.238	0.083	0.246	0.099	0.053	**<0.001**	0.061	**<0.001**	0.008	0.488
m = 3														
0.1	70.06	**<0.001**	0.487	0.076	0.528	0.062	0.517	0.073	0.041	**<0.001**	0.030	**<0.001**	-0.010	0.148
0.15	94.52	**<0.001**	0.442	0.096	0.502	0.090	0.504	0.107	0.060	**<0.001**	0.062	**<0.001**	0.002	0.970
0.2	92.44	**<0.001**	0.388	0.096	0.446	0.090	0.452	0.108	0.058	**<0.001**	0.064	**<0.001**	0.006	0.689
0.25	93.34	**<0.001**	0.340	0.092	0.395	0.083	0.402	0.101	0.055	**<0.001**	0.061	**<0.001**	0.007	0.611
0.3	94.93	**<0.001**	0.300	0.088	0.353	0.078	0.359	0.095	0.053	**<0.001**	0.059	**<0.001**	0.006	0.648
0.35	97.34	**<0.001**	0.266	0.085	0.317	0.075	0.322	0.089	0.052	**<0.001**	0.056	**<0.001**	0.005	0.764
0.4	98.18	**<0.001**	0.237	0.082	0.286	0.072	0.290	0.085	0.050	**<0.001**	0.053	**<0.001**	0.004	0.836
0.45	98.11	**<0.001**	0.211	0.078	0.259	0.070	0.262	0.082	0.048	**<0.001**	0.051	**<0.001**	0.004	0.823
0.5	98.05	**<0.001**	0.189	0.074	0.234	0.067	0.238	0.078	0.045	**<0.001**	0.049	**<0.001**	0.004	0.783
m = 4														
0.1	8.86	**<0.001**	0.377	0.045	0.383	0.043	0.369	0.050	0.006	**0.011**	-0.007	0.070	-0.014	**<0.001**
0.15	94.50	**<0.001**	0.386	0.074	0.432	0.062	0.425	0.074	0.046	**<0.001**	0.039	**<0.001**	-0.007	0.440
0.2	101.23	**<0.001**	0.351	0.085	0.407	0.080	0.406	0.093	0.055	**<0.001**	0.055	**<0.001**	0	0.998
0.25	96.17	**<0.001**	0.313	0.085	0.366	0.081	0.370	0.096	0.053	**<0.001**	0.057	**<0.001**	0.004	0.825
0.3	93	**<0.001**	0.279	0.081	0.328	0.077	0.334	0.091	0.049	**<0.001**	0.055	**<0.001**	0.006	0.646
0.35	94.25	**<0.001**	0.249	0.077	0.295	0.072	0.301	0.085	0.047	**<0.001**	0.052	**<0.001**	0.006	0.602
0.4	95.30	**<0.001**	0.223	0.072	0.267	0.067	0.272	0.079	0.044	**<0.001**	0.049	**<0.001**	0.005	0.622
0.45	95.05	**<0.001**	0.201	0.068	0.243	0.063	0.248	0.074	0.041	**<0.001**	0.046	**<0.001**	0.005	0.635
0.5	95.07	**<0.001**	0.182	0.065	0.221	0.059	0.226	0.070	0.039	**<0.001**	0.044	**<0.001**	0.005	0.612
m = 5														
0.1	48.65	**<0.001**	0.276	0.048	0.256	0.056	0.245	0.064	-0.020	**<0.001**	-0.031	**<0.001**	-0.011	**0.022**
0.15	55.71	**<0.001**	0.324	0.049	0.348	0.037	0.336	0.045	0.023	**<0.001**	0.011	**0.002**	-0.012	**0.002**
0.2	101.59	**<0.001**	0.315	0.067	0.359	0.058	0.352	0.067	0.044	**<0.001**	0.037	**<0.001**	-0.007	0.363
0.25	100.98	**<0.001**	0.290	0.075	0.338	0.069	0.336	0.080	0.048	**<0.001**	0.047	**<0.001**	-0.001	0.966
0.3	94.79	**<0.001**	0.262	0.075	0.309	0.070	0.311	0.082	0.047	**<0.001**	0.049	**<0.001**	0.002	0.928
0.35	94.20	**<0.001**	0.236	0.072	0.281	0.068	0.285	0.080	0.044	**<0.001**	0.048	**<0.001**	0.004	0.774
0.4	94.45	**<0.001**	0.213	0.068	0.255	0.065	0.260	0.076	0.042	**<0.001**	0.046	**<0.001**	0.004	0.686
0.45	94.46	**<0.001**	0.193	0.064	0.233	0.061	0.237	0.072	0.039	**<0.001**	0.044	**<0.001**	0.005	0.623
0.5	94.15	**<0.001**	0.176	0.061	0.213	0.057	0.217	0.067	0.037	**<0.001**	0.042	**<0.001**	0.005	0.573

F F-statistic, *SD* standard deviation, *MD* mean difference
Bold values indicate significant differences

Table S4 Approximate entropy in the medial-lateral direction as a function of *r* and *m* for a data length of *N*=600 (i.e. 30-seconds).

	One-way ANOVA		Descriptive statistics by group						Post-hoc					
			Young (Y)		Non-Fallers (NF)		Fallers (F)		NF - Y		F - Y		F - NF	
r	**F**	**p-value**	**Mean**	**SD**	**Mean**	**SD**	**Mean**	**SD**	**MD**	**p-value**	**MD**	**p-value**	**MD**	**p-value**
***m* = 2**														
0.1	9.49	**<0.001**	0.641	0.092	0.631	0.085	0.613	0.089	-0.010	0.059	-0.028	**<0.001**	-0.018	**0.028**
0.15	4.57	**0.010**	0.564	0.097	0.560	0.089	0.543	0.099	-0.004	0.656	-0.021	**0.007**	-0.017	0.051
0.2	2.35	0.095	0.489	0.104	0.489	0.098	0.473	0.109	0	0.996	-0.016	0.087	-0.016	0.120
0.25	1.77	0.170	0.421	0.107	0.423	0.102	0.407	0.113	0.002	0.927	-0.013	0.210	-0.015	0.154
0.3	1.47	0.230	0.362	0.106	0.364	0.102	0.350	0.111	0.002	0.939	-0.012	0.274	-0.014	0.211
0.35	1.33	0.265	0.312	0.101	0.313	0.097	0.301	0.106	0.001	0.964	-0.011	0.298	-0.012	0.252
0.4	1.15	0.318	0.270	0.094	0.271	0.091	0.260	0.098	0.001	0.991	-0.010	0.329	-0.011	0.319
0.45	1.02	0.361	0.235	0.086	0.235	0.083	0.226	0.091	0	0.997	-0.009	0.363	-0.009	0.371
0.5	0.94	0.392	0.206	0.078	0.206	0.076	0.199	0.083	0	0.999	-0.008	0.377	-0.008	0.425
***m* = 3**														
0.1	7.89	**<0.001**	0.497	0.065	0.492	0.068	0.477	0.072	-0.005	0.325	-0.020	**<0.001**	-0.015	**0.011**
0.15	7.34	**0.001**	0.457	0.082	0.449	0.079	0.434	0.083	-0.008	0.148	-0.023	**0.001**	-0.015	**0.046**
0.2	5.91	**0.003**	0.404	0.080	0.397	0.073	0.385	0.079	-0.007	0.164	-0.019	**0.003**	-0.012	0.112
0.25	3.91	**0.020**	0.358	0.076	0.354	0.069	0.343	0.076	-0.004	0.442	-0.015	**0.016**	-0.011	0.151
0.3	2.89	0.056	0.321	0.074	0.318	0.068	0.308	0.074	-0.003	0.759	-0.013	**0.043**	-0.010	0.153
0.35	2.13	0.119	0.287	0.072	0.286	0.066	0.276	0.073	-0.001	0.945	-0.011	0.100	-0.010	0.184
0.4	1.73	0.177	0.258	0.070	0.257	0.065	0.248	0.071	0	0.998	-0.009	0.166	-0.009	0.205
0.45	1.53	0.217	0.232	0.068	0.232	0.064	0.223	0.069	0	0.988	-0.008	0.227	-0.009	0.219
0.5	1.33	0.264	0.208	0.065	0.209	0.062	0.201	0.067	0.001	0.967	-0.007	0.294	-0.008	0.252
***m* = 4**														
0.1	2.87	0.057	0.378	0.038	0.381	0.041	0.373	0.042	0.003	0.361	-0.005	0.268	-0.007	0.050
0.15	5.09	**0.006**	0.395	0.062	0.393	0.064	0.380	0.069	-0.002	0.761	-0.015	**0.004**	-0.013	**0.025**
0.2	5.28	**0.005**	0.362	0.073	0.357	0.071	0.345	0.075	-0.005	0.322	-0.017	**0.004**	-0.012	0.082
0.25	5.23	**0.005**	0.325	0.072	0.319	0.066	0.308	0.072	-0.006	0.222	-0.016	**0.005**	-0.011	0.130
0.3	4.68	**0.009**	0.292	0.068	0.286	0.061	0.277	0.066	-0.005	0.249	-0.014	**0.009**	-0.009	0.168
0.35	3.94	**0.020**	0.263	0.064	0.259	0.057	0.251	0.062	-0.004	0.352	-0.013	**0.018**	-0.008	0.193
0.4	3.25	**0.039**	0.239	0.060	0.235	0.054	0.228	0.059	-0.003	0.505	-0.011	**0.033**	-0.008	0.209
0.45	2.45	0.087	0.217	0.057	0.215	0.052	0.208	0.057	-0.002	0.730	-0.009	0.070	-0.007	0.232
0.5	1.90	0.150	0.198	0.055	0.197	0.050	0.190	0.055	-0.001	0.870	-0.008	0.125	-0.006	0.269
***m* = 5**														
0.1	1.18	0.309	0.271	0.046	0.273	0.042	0.275	0.041	0.003	0.428	0.004	0.458	0.001	0.933
0.15	3.88	**0.021**	0.331	0.040	0.332	0.043	0.323	0.048	0.001	0.860	-0.008	**0.033**	-0.009	**0.017**
0.2	4.24	**0.014**	0.325	0.057	0.323	0.058	0.312	0.063	-0.002	0.702	-0.013	**0.010**	-0.010	0.059
0.25	4.02	**0.018**	0.299	0.064	0.296	0.062	0.286	0.066	-0.003	0.596	-0.013	**0.013**	-0.010	0.092
0.3	4.24	**0.015**	0.273	0.063	0.269	0.059	0.259	0.063	-0.004	0.376	-0.013	**0.012**	-0.009	0.145
0.35	4.20	**0.015**	0.248	0.061	0.243	0.055	0.235	0.060	-0.004	0.301	-0.012	**0.014**	-0.008	0.191
0.4	4	**0.018**	0.226	0.058	0.221	0.052	0.214	0.056	-0.004	0.282	-0.011	**0.019**	-0.007	0.236
0.45	3.21	**0.041**	0.206	0.054	0.203	0.048	0.196	0.053	-0.003	0.419	-0.010	**0.038**	-0.006	0.268
0.5	2.70	0.067	0.188	0.051	0.186	0.046	0.180	0.050	-0.003	0.541	-0.008	0.059	-0.006	0.288

F F-statistic, *SD* standard deviation, *MD* mean difference
Bold values indicate significant differences

Table S5 Sample entropy in the medial-lateral direction as a function of *r* and *m* for a data length of *N*=1200 (i.e. 60-seconds).

	One-way ANOVA		Descriptive statistics by group						Post-hoc					
			Young (Y)		Non-Fallers (NF)		Fallers (F)		NF - Y		F - Y		F - NF	
r	**F**	**p-value**	**Mean**	**SD**	**Mean**	**SD**	**Mean**	**SD**	**MD**	**p-value**	**MD**	**p-value**	**MD**	**p-value**
***m* = 2**														
0.1	0.91	0.403	0.615	0.138	0.619	0.125	0.605	0.131	0.004	0.831	-0.010	0.562	-0.014	0.370
0.15	1.26	0.283	0.476	0.119	0.484	0.109	0.472	0.115	0.008	0.358	-0.004	0.905	-0.012	0.410
0.2	1.45	0.236	0.384	0.110	0.392	0.102	0.382	0.107	0.008	0.257	-0.002	0.975	-0.010	0.453
0.25	1.40	0.247	0.316	0.100	0.324	0.094	0.315	0.098	0.008	0.265	-0.001	0.981	-0.009	0.476
0.3	1.21	0.297	0.265	0.090	0.272	0.086	0.264	0.089	0.006	0.321	-0.001	0.978	-0.008	0.513
0.35	1.04	0.355	0.226	0.080	0.231	0.077	0.224	0.080	0.005	0.386	-0.001	0.974	-0.006	0.550
0.4	0.88	0.416	0.194	0.071	0.198	0.068	0.193	0.072	0.004	0.453	-0.001	0.972	-0.005	0.592
0.45	0.74	0.476	0.169	0.063	0.173	0.061	0.168	0.064	0.003	0.509	-0.001	0.978	-0.004	0.644
0.5	0.64	0.530	0.149	0.057	0.152	0.054	0.148	0.058	0.003	0.559	-0.001	0.982	-0.004	0.689
***m* = 3**														
0.1	1.30	0.273	0.551	0.143	0.555	0.136	0.537	0.141	0.004	0.870	-0.014	0.369	-0.018	0.243
0.15	0.92	0.397	0.420	0.107	0.423	0.099	0.412	0.103	0.003	0.811	-0.008	0.571	-0.011	0.364
0.2	0.84	0.433	0.343	0.090	0.347	0.082	0.339	0.086	0.004	0.660	-0.005	0.764	-0.008	0.442
0.25	0.92	0.397	0.290	0.081	0.295	0.073	0.288	0.077	0.004	0.513	-0.003	0.881	-0.007	0.479
0.3	0.96	0.384	0.250	0.074	0.255	0.068	0.249	0.071	0.004	0.445	-0.002	0.944	-0.006	0.528
0.35	0.97	0.379	0.218	0.068	0.222	0.063	0.217	0.065	0.004	0.426	-0.001	0.959	-0.005	0.545
0.4	0.93	0.396	0.192	0.063	0.196	0.059	0.191	0.061	0.004	0.437	-0.001	0.967	-0.005	0.568
0.45	0.87	0.420	0.170	0.058	0.174	0.055	0.169	0.056	0.003	0.454	-0.001	0.974	-0.004	0.599
0.5	0.79	0.456	0.152	0.054	0.155	0.051	0.151	0.052	0.003	0.488	-0.001	0.978	-0.004	0.630
***m* = 4**														
0.1	1.64	0.194	0.525	0.145	0.532	0.139	0.512	0.143	0.007	0.589	-0.013	0.444	-0.020	0.175
0.15	1.23	0.292	0.398	0.108	0.402	0.102	0.389	0.106	0.004	0.739	-0.009	0.488	-0.013	0.262
0.2	0.94	0.389	0.324	0.089	0.326	0.082	0.317	0.085	0.003	0.778	-0.006	0.590	-0.009	0.360
0.25	0.70	0.495	0.274	0.076	0.276	0.069	0.269	0.073	0.003	0.763	-0.004	0.739	-0.007	0.482
0.3	0.66	0.519	0.237	0.068	0.239	0.061	0.234	0.064	0.003	0.709	-0.003	0.824	-0.006	0.534
0.35	0.65	0.524	0.208	0.062	0.211	0.056	0.206	0.059	0.003	0.659	-0.002	0.882	-0.005	0.572
0.4	0.65	0.525	0.185	0.057	0.187	0.052	0.183	0.054	0.003	0.615	-0.002	0.927	-0.004	0.609
0.45	0.62	0.537	0.165	0.053	0.167	0.048	0.164	0.050	0.002	0.598	-0.001	0.957	-0.004	0.651
0.5	0.59	0.556	0.148	0.049	0.151	0.045	0.148	0.047	0.002	0.600	-0.001	0.972	-0.003	0.686
***m* = 5**														
0.1	1.88	0.153	0.501	0.142	0.509	0.137	0.489	0.140	0.008	0.443	-0.012	0.491	-0.020	0.151
0.15	1.44	0.238	0.382	0.106	0.387	0.101	0.373	0.105	0.005	0.640	-0.009	0.483	-0.014	0.216
0.2	1.20	0.301	0.312	0.087	0.315	0.0820	0.305	0.085	0.003	0.736	-0.007	0.503	-0.010	0.272
0.25	0.89	0.411	0.264	0.075	0.267	0.0690	0.259	0.072	0.003	0.769	-0.005	0.628	-0.008	0.385
0.3	0.71	0.491	0.229	0.067	0.231	0.0610	0.225	0.063	0.002	0.770	-0.004	0.727	-0.006	0.475
0.35	0.63	0.533	0.201	0.060	0.203	0.0540	0.198	0.057	0.002	0.759	-0.003	0.789	-0.005	0.529
0.4	0.54	0.581	0.179	0.055	0.181	0.0500	0.177	0.052	0.002	0.737	-0.002	0.866	-0.004	0.602
0.45	0.51	0.600	0.160	0.050	0.162	0.0460	0.159	0.047	0.002	0.708	-0.001	0.916	-0.003	0.650
0.5	0.46	0.634	0.144	0.047	0.146	0.0430	0.143	0.044	0.002	0.703	-0.001	0.953	-0.003	0.710

F F-statistic, *SD* standard deviation, *MD* mean difference

Bold values indicate significant differences

Table S6 Sample entropy in the anterior-posterior direction as a function of *r* and *m* for a data length of *N*=600 (i.e. 30-seconds).

	One-way ANOVA		Descriptive statistics by group						Post-hoc					
			Young (Y)		Non-Fallers (NF)		Fallers (F)		NF - Y		F - Y		F - NF	
r	**F**	**p-value**	**Mean**	**SD**	**Mean**	**SD**	**Mean**	**SD**	**MD**	**p-value**	**MD**	**p-value**	**MD**	**p-value**
m = 2														
0.1	96.27	**<0.001**	0.622	0.165	0.724	0.156	0.737	0.187	0.102	**<0.001**	0.115	**<0.001**	0.013	0.565
0.15	107.59	**<0.001**	0.484	0.137	0.572	0.122	0.580	0.148	0.088	**<0.001**	0.096	**<0.001**	0.009	0.694
0.2	111.29	**<0.001**	0.394	0.124	0.476	0.112	0.481	0.133	0.082	**<0.001**	0.087	**<0.001**	0.005	0.871
0.25	111.62	**<0.001**	0.328	0.114	0.404	0.105	0.408	0.124	0.076	**<0.001**	0.080	**<0.001**	0.004	0.894
0.3	109.64	**<0.001**	0.277	0.104	0.347	0.099	0.351	0.116	0.070	**<0.001**	0.074	**<0.001**	0.004	0.896
0.35	107.43	**<0.001**	0.237	0.095	0.301	0.092	0.305	0.108	0.063	**<0.001**	0.067	**<0.001**	0.004	0.845
0.4	105.38	**<0.001**	0.206	0.085	0.262	0.085	0.267	0.099	0.057	**<0.001**	0.061	**<0.001**	0.005	0.787
0.45	103.19	**<0.001**	0.180	0.077	0.230	0.077	0.235	0.091	0.051	**<0.001**	0.056	**<0.001**	0.005	0.719
0.5	101.31	**<0.001**	0.159	0.069	0.204	0.070	0.209	0.083	0.045	**<0.001**	0.050	**<0.001**	0.005	0.667
m = 3														
0.1	98.14	**<0.001**	0.567	0.175	0.679	0.172	0.688	0.204	0.112	**<0.001**	0.121	**<0.001**	0.009	0.802
0.15	105.90	**<0.001**	0.432	0.132	0.519	0.131	0.531	0.158	0.087	**<0.001**	0.099	**<0.001**	0.012	0.508
0.2	106.88	**<0.001**	0.354	0.109	0.426	0.107	0.435	0.129	0.072	**<0.001**	0.081	**<0.001**	0.009	0.530
0.25	107.26	**<0.001**	0.300	0.095	0.362	0.091	0.369	0.110	0.062	**<0.001**	0.070	**<0.001**	0.007	0.579
0.3	107.27	**<0.001**	0.259	0.086	0.315	0.081	0.321	0.098	0.056	**<0.001**	0.062	**<0.001**	0.006	0.669
0.35	107.29	**<0.001**	0.226	0.079	0.278	0.074	0.282	0.088	0.052	**<0.001**	0.056	**<0.001**	0.004	0.780
0.4	106.56	**<0.001**	0.200	0.073	0.248	0.069	0.251	0.082	0.048	**<0.001**	0.052	**<0.001**	0.003	0.815
0.45	105.75	**<0.001**	0.178	0.068	0.222	0.065	0.225	0.076	0.045	**<0.001**	0.048	**<0.001**	0.003	0.820
0.5	103.89	**<0.001**	0.159	0.063	0.200	0.061	0.203	0.071	0.041	**<0.001**	0.044	**<0.001**	0.003	0.809
m = 4														
0.1	96.77	**<0.001**	0.538	0.176	0.651	0.174	0.657	0.205	0.113	**<0.001**	0.119	**<0.001**	0.006	0.909
0.15	107.22	**<0.001**	0.410	0.132	0.499	0.132	0.508	0.159	0.089	**<0.001**	0.098	**<0.001**	0.009	0.687
0.2	108.88	**<0.001**	0.336	0.108	0.410	0.109	0.417	0.132	0.074	**<0.001**	0.081	**<0.001**	0.007	0.685
0.25	106.78	**<0.001**	0.285	0.093	0.347	0.093	0.354	0.112	0.062	**<0.001**	0.070	**<0.001**	0.007	0.579
0.3	104.93	**<0.001**	0.247	0.082	0.301	0.081	0.308	0.098	0.054	**<0.001**	0.061	**<0.001**	0.007	0.566
0.35	104.19	**<0.001**	0.217	0.074	0.265	0.073	0.271	0.087	0.049	**<0.001**	0.054	**<0.001**	0.006	0.613
0.4	104.08	**<0.001**	0.192	0.068	0.237	0.066	0.241	0.078	0.044	**<0.001**	0.049	**<0.001**	0.005	0.644
0.45	103.50	**<0.001**	0.172	0.063	0.213	0.061	0.217	0.072	0.041	**<0.001**	0.045	**<0.001**	0.004	0.682
0.5	102.30	**<0.001**	0.155	0.058	0.192	0.056	0.196	0.066	0.038	**<0.001**	0.041	**<0.001**	0.004	0.693
m = 5														
0.1	75.78	**<0.001**	0.509	0.192	0.615	0.176	0.617	0.211	0.106	**<0.001**	0.109	**<0.001**	0.002	0.987
0.15	103.92	**<0.001**	0.389	0.128	0.475	0.128	0.480	0.152	0.086	**<0.001**	0.091	**<0.001**	0.005	0.874
0.2	107.87	**<0.001**	0.321	0.105	0.393	0.105	0.398	0.127	0.072	**<0.001**	0.077	**<0.001**	0.005	0.825
0.25	106.37	**<0.001**	0.274	0.091	0.335	0.091	0.341	0.109	0.061	**<0.001**	0.067	**<0.001**	0.006	0.703
0.3	104.62	**<0.001**	0.238	0.081	0.292	0.080	0.297	0.096	0.053	**<0.001**	0.059	**<0.001**	0.006	0.654
0.35	104.18	**<0.001**	0.210	0.073	0.258	0.072	0.263	0.086	0.048	**<0.001**	0.053	**<0.001**	0.005	0.647
0.4	103.06	**<0.001**	0.187	0.066	0.230	0.066	0.235	0.077	0.043	**<0.001**	0.048	**<0.001**	0.005	0.655
0.45	101.96	**<0.001**	0.168	0.061	0.207	0.060	0.211	0.070	0.039	**<0.001**	0.044	**<0.001**	0.004	0.652
0.5	100.66	**<0.001**	0.151	0.056	0.187	0.055	0.191	0.065	0.036	**<0.001**	0.040	**<0.001**	0.004	0.645

F F-statistic, *SD* standard deviation, *MD* mean difference
Bold values indicate significant differences

Table S7 Sample entropy in the medial-lateral direction as a function of *r* and *m* for a data length of *N*=600 (i.e. 30-seconds).

	One-way ANOVA		Descriptive statistics by group						Post-hoc					
			Young (Y)		Non-Fallers (NF)		Fallers (F)		NF - Y		F - Y		F - NF	
r	**F**	**p-value**	**Mean**	**SD**	**Mean**	**SD**	**Mean**	**SD**	**MD**	**p-value**	**MD**	**p-value**	**MD**	**p-value**
m = 2														
0.1	4.27	**0.014**	0.658	0.145	0.651	0.133	0.627	0.139	-0.007	0.581	-0.031	**0.010**	-0.024	0.079
0.15	2.43	0.089	0.516	0.119	0.516	0.110	0.498	0.119	0	1	-0.019	0.083	-0.018	0.106
0.2	1.77	0.170	0.424	0.109	0.427	0.103	0.411	0.112	0.003	0.875	-0.013	0.234	-0.016	0.148
0.25	1.62	0.197	0.355	0.102	0.359	0.098	0.345	0.106	0.004	0.703	-0.010	0.367	-0.014	0.170
0.3	1.52	0.220	0.302	0.094	0.306	0.091	0.293	0.098	0.004	0.676	-0.009	0.423	-0.013	0.194
0.35	1.40	0.246	0.259	0.086	0.263	0.084	0.251	0.090	0.004	0.690	-0.008	0.456	-0.011	0.219
0.4	1.25	0.286	0.225	0.078	0.228	0.077	0.218	0.082	0.003	0.740	-0.007	0.479	-0.010	0.256
0.45	1.12	0.327	0.197	0.071	0.199	0.069	0.191	0.074	0.002	0.772	-0.006	0.511	-0.008	0.295
0.5	1.01	0.363	0.174	0.064	0.176	0.062	0.169	0.067	0.002	0.813	-0.005	0.527	-0.007	0.330
m = 3														
0.1	4.34	**0.013**	0.593	0.158	0.588	0.150	0.559	0.152	-0.006	0.743	-0.034	**0.009**	-0.029	**0.049**
0.15	3.71	**0.025**	0.453	0.115	0.448	0.107	0.430	0.111	-0.005	0.601	-0.023	**0.018**	-0.017	0.116
0.2	3.01	**0.049**	0.373	0.093	0.370	0.086	0.356	0.090	-0.003	0.733	-0.017	**0.038**	-0.013	0.147
0.25	2.29	0.101	0.318	0.082	0.317	0.075	0.305	0.079	-0.001	0.945	-0.013	0.085	-0.011	0.159
0.3	1.94	0.145	0.277	0.075	0.278	0.069	0.267	0.074	0	0.996	-0.010	0.146	-0.011	0.154
0.35	1.60	0.203	0.244	0.070	0.245	0.065	0.236	0.069	0.001	0.947	-0.008	0.239	-0.009	0.189
0.4	1.43	0.240	0.217	0.065	0.218	0.061	0.210	0.065	0.002	0.880	-0.007	0.321	-0.008	0.212
0.45	1.32	0.266	0.194	0.061	0.195	0.058	0.188	0.062	0.002	0.817	-0.006	0.395	-0.008	0.234
0.5	1.24	0.290	0.174	0.057	0.176	0.054	0.169	0.058	0.002	0.784	-0.005	0.452	-0.007	0.258
m = 4														
0.1	3.50	**0.030**	0.562	0.160	0.560	0.153	0.531	0.156	-0.002	0.980	-0.031	**0.026**	-0.029	**0.048**
0.15	3.37	**0.035**	0.428	0.117	0.424	0.110	0.405	0.115	-0.004	0.784	-0.022	**0.026**	-0.019	0.098
0.2	3.18	**0.042**	0.350	0.094	0.346	0.088	0.333	0.091	-0.004	0.673	-0.017	**0.032**	-0.013	0.147
0.25	2.86	0.058	0.298	0.080	0.295	0.073	0.284	0.077	-0.003	0.717	-0.014	**0.045**	-0.011	0.173
0.3	2.45	0.087	0.260	0.070	0.258	0.064	0.249	0.068	-0.002	0.825	-0.011	0.069	-0.009	0.190
0.35	2.14	0.118	0.230	0.064	0.229	0.058	0.220	0.062	-0.001	0.919	-0.010	0.098	-0.008	0.196
0.4	1.76	0.172	0.206	0.059	0.205	0.054	0.198	0.057	-0.001	0.975	-0.008	0.152	-0.007	0.228
0.45	1.48	0.228	0.185	0.055	0.185	0.050	0.179	0.053	0	1	-0.007	0.217	-0.007	0.255
0.5	1.29	0.276	0.168	0.051	0.168	0.047	0.162	0.050	0	0.993	-0.006	0.283	-0.006	0.280
m = 5														
0.1	2.58	0.076	0.529	0.160	0.530	0.157	0.504	0.160	0.001	0.991	-0.026	0.079	-0.027	0.081
0.15	2.92	0.054	0.406	0.113	0.403	0.108	0.385	0.114	-0.002	0.907	-0.020	**0.043**	-0.018	0.103
0.2	2.78	0.062	0.334	0.091	0.331	0.086	0.318	0.092	-0.003	0.758	-0.016	**0.048**	-0.013	0.167
0.25	2.70	0.067	0.285	0.078	0.283	0.073	0.272	0.077	-0.003	0.775	-0.013	0.053	-0.011	0.172
0.3	2.48	0.084	0.249	0.069	0.247	0.064	0.238	0.067	-0.002	0.827	-0.011	0.067	-0.009	0.183
0.35	2.29	0.102	0.221	0.062	0.219	0.057	0.211	0.060	-0.002	0.837	-0.010	0.082	-0.008	0.21
0.4	2.02	0.134	0.198	0.057	0.196	0.052	0.190	0.055	-0.001	0.862	-0.008	0.110	-0.007	0.249
0.45	1.66	0.190	0.179	0.052	0.178	0.048	0.172	0.051	-0.001	0.926	-0.007	0.163	-0.006	0.288
0.5	1.49	0.225	0.162	0.049	0.161	0.044	0.156	0.047	-0.001	0.965	-0.006	0.199	-0.005	0.301

F F-statistic, *SD* standard deviation, *MD* mean difference
Bold values indicate significant differences

Table S8 Sample entropy in the anterior-posterior direction as a function of *r* and *m* for a data length of *N*=1200 (i.e. 60-seconds): eyes open – rigid surface (OR)

	**One-way ANOVA**		**Summary statistics by group**						**Post-hoc**					
			**Young (Y)**		**Non-Fallers (NF)**		**Fallers (F)**		**NF - Y**		**F - Y**		**F - NF**	
r	**F**	**p-value**	**Mean**	**SD**	**Mean**	**SD**	**Mean**	**SD**	**MD**	**p-value**	**MD**	**p-value**	**MD**	**p-value**
m = 2														
0.1	25.84	**<0.001**	0.540	0.156	0.640	0.157	0.662	0.190	0.100	**<0.001**	0.121	**<0.001**	0.022	0.663
0.15	30.58	**<0.001**	0.399	0.133	0.492	0.129	0.509	0.161	0.092	**<0.001**	0.109	**<0.001**	0.017	0.703
0.2	31.51	**<0.001**	0.310	0.118	0.394	0.117	0.411	0.148	0.084	**<0.001**	0.101	**<0.001**	0.017	0.628
0.25	31.25	**<0.001**	0.249	0.104	0.323	0.107	0.342	0.138	0.074	**<0.001**	0.093	**<0.001**	0.019	0.505
0.3	30.82	**<0.001**	0.204	0.091	0.269	0.096	0.288	0.127	0.064	**<0.001**	0.084	**<0.001**	0.020	0.405
0.35	30.26	**<0.001**	0.171	0.079	0.227	0.086	0.247	0.115	0.056	**<0.001**	0.075	**<0.001**	0.019	0.325
0.4	29.78	**<0.001**	0.146	0.070	0.195	0.077	0.214	0.104	0.049	**<0.001**	0.067	**<0.001**	0.019	0.264
0.45	29.52	**<0.001**	0.127	0.061	0.169	0.068	0.187	0.094	0.043	**<0.001**	0.060	**<0.001**	0.018	0.224
0.5	29.39	**<0.001**	0.111	0.054	0.149	0.061	0.165	0.085	0.038	**<0.001**	0.054	**<0.001**	0.016	0.196
m = 3														
0.1	24.08	**<0.001**	0.488	0.152	0.587	0.170	0.612	0.208	0.098	**<0.001**	0.124	**<0.001**	0.025	0.593
0.15	28.21	**<0.001**	0.363	0.114	0.442	0.127	0.463	0.155	0.079	**<0.001**	0.100	**<0.001**	0.021	0.538
0.2	30.17	**<0.001**	0.289	0.096	0.358	0.103	0.374	0.127	0.068	**<0.001**	0.085	**<0.001**	0.016	0.565
0.25	31.34	**<0.001**	0.238	0.086	0.300	0.089	0.314	0.112	0.062	**<0.001**	0.076	**<0.001**	0.014	0.569
0.3	32.03	**<0.001**	0.200	0.078	0.257	0.080	0.270	0.102	0.056	**<0.001**	0.069	**<0.001**	0.013	0.570
0.35	32.05	**<0.001**	0.171	0.071	0.222	0.073	0.235	0.094	0.051	**<0.001**	0.064	**<0.001**	0.012	0.538
0.4	31.88	**<0.001**	0.148	0.064	0.194	0.068	0.207	0.087	0.047	**<0.001**	0.059	**<0.001**	0.012	0.479
0.45	31.47	**<0.001**	0.129	0.059	0.171	0.062	0.184	0.081	0.042	**<0.001**	0.054	**<0.001**	0.012	0.421
0.5	31.06	**<0.001**	0.114	0.053	0.152	0.057	0.164	0.075	0.038	**<0.001**	0.050	**<0.001**	0.012	0.364
m = 4														
0.1	25.47	**<0.001**	0.463	0.151	0.566	0.171	0.588	0.214	0.103	**<0.001**	0.124	**<0.001**	0.022	0.684
0.15	28.46	**<0.001**	0.343	0.111	0.424	0.130	0.442	0.158	0.081	**<0.001**	0.099	**<0.001**	0.018	0.613
0.2	29.72	**<0.001**	0.275	0.092	0.341	0.103	0.358	0.127	0.066	**<0.001**	0.083	**<0.001**	0.017	0.530
0.25	30.52	**<0.001**	0.228	0.080	0.286	0.087	0.301	0.109	0.057	**<0.001**	0.073	**<0.001**	0.015	0.489
0.3	31.34	**<0.001**	0.194	0.071	0.245	0.076	0.258	0.096	0.051	**<0.001**	0.065	**<0.001**	0.013	0.504
0.35	31.83	**<0.001**	0.167	0.064	0.214	0.069	0.226	0.087	0.047	**<0.001**	0.059	**<0.001**	0.012	0.498
0.4	32.05	**<0.001**	0.145	0.059	0.188	0.062	0.200	0.079	0.043	**<0.001**	0.054	**<0.001**	0.011	0.486
0.45	32.04	**<0.001**	0.128	0.054	0.167	0.057	0.178	0.073	0.039	**<0.001**	0.050	**<0.001**	0.011	0.463
0.5	31.97	**<0.001**	0.113	0.050	0.150	0.053	0.160	0.067	0.036	**<0.001**	0.046	**<0.001**	0.010	0.435
m = 5														
0.1	24.20	**<0.001**	0.444	0.148	0.543	0.167	0.561	0.205	0.099	**<0.001**	0.116	**<0.001**	0.018	0.767
0.15	28.85	**<0.001**	0.331	0.109	0.410	0.126	0.428	0.156	0.079	**<0.001**	0.097	**<0.001**	0.018	0.605
0.2	29.91	**<0.001**	0.265	0.089	0.330	0.100	0.348	0.128	0.065	**<0.001**	0.083	**<0.001**	0.018	0.467
0.25	30.55	**<0.001**	0.221	0.078	0.277	0.086	0.294	0.109	0.056	**<0.001**	0.072	**<0.001**	0.016	0.425
0.3	31.29	**<0.001**	0.188	0.069	0.238	0.075	0.253	0.096	0.050	**<0.001**	0.064	**<0.001**	0.015	0.421
0.35	31.44	**<0.001**	0.163	0.062	0.208	0.067	0.221	0.085	0.045	**<0.001**	0.058	**<0.001**	0.013	0.426
0.4	31.80	**<0.001**	0.143	0.057	0.184	0.061	0.195	0.077	0.041	**<0.001**	0.053	**<0.001**	0.012	0.422
0.45	31.90	**<0.001**	0.126	0.052	0.163	0.055	0.175	0.070	0.037	**<0.001**	0.049	**<0.001**	0.011	0.389
0.5	32.05	**<0.001**	0.112	0.048	0.146	0.051	0.157	0.065	0.034	**<0.001**	0.045	**<0.001**	0.011	0.378

F F-statistic, *SD* standard deviation, *MD* mean difference
Bold values indicate significant differences

Table S9 Sample entropy in the anterior-posterior direction as a function of *r* and *m* for a data length of *N*=1200 (i.e. 60-seconds): eyes closed – rigid surface (CR)

	**One-way ANOVA**		**Summary statistics by group**						**Post-hoc**					
			**Young (Y)**		**Non-Fallers (NF)**		**Fallers (F)**		**NF - Y**		**F - Y**		**F - NF**	
r	**F**	**p-value**	**Mean**	**SD**	**Mean**	**SD**	**Mean**	**SD**	**MD**	**p-value**	**MD**	**p-value**	**MD**	**p-value**
m = 2														
0.1	38.88	**<0.001**	0.542	0.167	0.673	0.157	0.694	0.212	0.131	**<0.001**	0.152	**<0.001**	0.021	0.706
0.15	44.38	**<0.001**	0.401	0.142	0.520	0.129	0.532	0.172	0.119	**<0.001**	0.132	**<0.001**	0.013	0.830
0.2	45.93	**<0.001**	0.312	0.125	0.421	0.119	0.433	0.153	0.108	**<0.001**	0.121	**<0.001**	0.012	0.807
0.25	46.09	**<0.001**	0.251	0.110	0.348	0.111	0.362	0.140	0.097	**<0.001**	0.111	**<0.001**	0.013	0.732
0.3	45.55	**<0.001**	0.207	0.096	0.293	0.103	0.307	0.127	0.086	**<0.001**	0.100	**<0.001**	0.014	0.649
0.35	44.90	**<0.001**	0.174	0.084	0.250	0.094	0.264	0.115	0.076	**<0.001**	0.090	**<0.001**	0.015	0.565
0.4	44.32	**<0.001**	0.149	0.074	0.215	0.085	0.230	0.103	0.066	**<0.001**	0.081	**<0.001**	0.014	0.492
0.45	43.86	**<0.001**	0.129	0.065	0.188	0.076	0.201	0.093	0.058	**<0.001**	0.072	**<0.001**	0.014	0.450
0.5	43.55	**<0.001**	0.114	0.057	0.165	0.069	0.178	0.083	0.052	**<0.001**	0.065	**<0.001**	0.013	0.418
m = 3														
0.1	36.82	**<0.001**	0.490	0.162	0.622	0.173	0.648	0.236	0.131	**<0.001**	0.157	**<0.001**	0.026	0.613
0.15	41.47	**<0.001**	0.365	0.123	0.469	0.128	0.488	0.174	0.104	**<0.001**	0.124	**<0.001**	0.019	0.610
0.2	43.70	**<0.001**	0.291	0.104	0.380	0.104	0.395	0.140	0.089	**<0.001**	0.104	**<0.001**	0.015	0.644
0.25	44.96	**<0.001**	0.240	0.092	0.320	0.090	0.332	0.120	0.079	**<0.001**	0.092	**<0.001**	0.012	0.682
0.3	45.53	**<0.001**	0.202	0.083	0.275	0.082	0.285	0.107	0.072	**<0.001**	0.083	**<0.001**	0.011	0.710
0.35	45.61	**<0.001**	0.173	0.075	0.239	0.076	0.249	0.097	0.066	**<0.001**	0.076	**<0.001**	0.010	0.682
0.4	45.09	**<0.001**	0.150	0.069	0.210	0.071	0.220	0.089	0.060	**<0.001**	0.070	**<0.001**	0.010	0.653
0.45	44.64	**<0.001**	0.132	0.062	0.186	0.066	0.196	0.082	0.055	**<0.001**	0.064	**<0.001**	0.010	0.619
0.5	44.23	**<0.001**	0.116	0.057	0.166	0.061	0.175	0.075	0.050	**<0.001**	0.059	**<0.001**	0.009	0.586
m = 4														
0.1	37.73	**<0.001**	0.466	0.162	0.602	0.175	0.623	0.244	0.136	**<0.001**	0.157	**<0.001**	0.021	0.720
0.15	41.48	**<0.001**	0.345	0.120	0.450	0.130	0.470	0.182	0.105	**<0.001**	0.125	**<0.001**	0.020	0.609
0.2	43.35	**<0.001**	0.277	0.099	0.364	0.105	0.380	0.143	0.087	**<0.001**	0.103	**<0.001**	0.016	0.594
0.25	44.04	**<0.001**	0.230	0.086	0.305	0.089	0.319	0.120	0.075	**<0.001**	0.089	**<0.001**	0.014	0.590
0.3	44.66	**<0.001**	0.196	0.077	0.262	0.078	0.274	0.104	0.067	**<0.001**	0.079	**<0.001**	0.012	0.607
0.35	44.78	**<0.001**	0.169	0.069	0.229	0.070	0.240	0.092	0.060	**<0.001**	0.071	**<0.001**	0.011	0.611
0.4	44.63	**<0.001**	0.147	0.063	0.202	0.064	0.212	0.083	0.055	**<0.001**	0.064	**<0.001**	0.009	0.634
0.45	44.14	**<0.001**	0.130	0.058	0.180	0.059	0.189	0.076	0.050	**<0.001**	0.059	**<0.001**	0.009	0.629
0.5	43.79	**<0.001**	0.116	0.053	0.162	0.055	0.170	0.070	0.046	**<0.001**	0.054	**<0.001**	0.008	0.615
m = 5														
0.1	38.93	**<0.001**	0.444	0.156	0.576	0.170	0.614	0.264	0.131	**<0.001**	0.170	**<0.001**	0.039	0.340
0.15	42.01	**<0.001**	0.332	0.116	0.434	0.126	0.455	0.183	0.102	**<0.001**	0.123	**<0.001**	0.021	0.552
0.2	44.02	**<0.001**	0.267	0.096	0.353	0.103	0.369	0.143	0.086	**<0.001**	0.102	**<0.001**	0.016	0.601
0.25	44.44	**<0.001**	0.223	0.083	0.296	0.087	0.310	0.119	0.073	**<0.001**	0.087	**<0.001**	0.014	0.589
0.3	44.47	**<0.001**	0.190	0.074	0.255	0.077	0.267	0.103	0.065	**<0.001**	0.077	**<0.001**	0.012	0.578
0.35	44.65	**<0.001**	0.165	0.067	0.223	0.069	0.234	0.091	0.058	**<0.001**	0.069	**<0.001**	0.011	0.574
0.4	44.20	**<0.001**	0.144	0.061	0.197	0.062	0.207	0.082	0.053	**<0.001**	0.062	**<0.001**	0.010	0.590
0.45	43.84	**<0.001**	0.128	0.056	0.176	0.057	0.185	0.074	0.048	**<0.001**	0.057	**<0.001**	0.009	0.599
0.5	43.36	**<0.001**	0.114	0.051	0.158	0.053	0.166	0.068	0.044	**<0.001**	0.052	**<0.001**	0.008	0.589

F F-statistic, *SD* standard deviation, *MD* mean difference
Bold values indicate significant differences

Table S10 Sample entropy in the anterior-posterior direction as a function of *r* and *m* for a data length of *N*=1200 (i.e. 60-seconds): eyes open – foam surface (OF)

	One-way ANOVA		Summary statistics by group						Post-hoc					
			Young (Y)		Non-Fallers (NF)		Fallers (F)		NF - Y		F - Y		F - NF	
r	F	p-value	Mean	SD	Mean	SD	Mean	SD	MD	p-value	MD	p-value	MD	p-value
***m* = 2**														
0.1	70.99	**<0.001**	0.585	0.131	0.708	0.108	0.754	0.142	0.123	**<0.001**	0.169	**<0.001**	0.046	**0.047**
0.15	76.61	**<0.001**	0.457	0.113	0.566	0.086	0.599	0.106	0.109	**<0.001**	0.142	**<0.001**	0.033	0.104
0.2	76.84	**<0.001**	0.369	0.106	0.471	0.080	0.499	0.094	0.102	**<0.001**	0.129	**<0.001**	0.027	0.165
0.25	76.30	**<0.001**	0.304	0.097	0.399	0.077	0.424	0.088	0.095	**<0.001**	0.120	**<0.001**	0.025	0.173
0.3	74.13	**<0.001**	0.254	0.088	0.340	0.073	0.364	0.084	0.086	**<0.001**	0.110	**<0.001**	0.024	0.163
0.35	72.06	**<0.001**	0.216	0.079	0.293	0.068	0.314	0.079	0.077	**<0.001**	0.099	**<0.001**	0.022	0.161
0.4	70.07	**<0.001**	0.186	0.071	0.254	0.063	0.274	0.073	0.069	**<0.001**	0.088	**<0.001**	0.020	0.155
0.45	68.05	**<0.001**	0.162	0.063	0.222	0.057	0.240	0.067	0.061	**<0.001**	0.079	**<0.001**	0.018	0.151
0.5	66.23	**<0.001**	0.142	0.056	0.196	0.052	0.212	0.061	0.054	**<0.001**	0.070	**<0.001**	0.016	0.159
***m* = 3**														
0.1	68.71	**<0.001**	0.530	0.145	0.668	0.124	0.714	0.163	0.138	**<0.001**	0.184	**<0.001**	0.046	0.092
0.15	70.62	**<0.001**	0.408	0.109	0.514	0.097	0.552	0.126	0.106	**<0.001**	0.144	**<0.001**	0.039	0.056
0.2	71.92	**<0.001**	0.335	0.090	0.421	0.078	0.453	0.099	0.087	**<0.001**	0.118	**<0.001**	0.032	0.054
0.25	73.93	**<0.001**	0.282	0.079	0.359	0.066	0.385	0.084	0.077	**<0.001**	0.103	**<0.001**	0.026	0.067
0.3	74.18	**<0.001**	0.242	0.072	0.312	0.059	0.335	0.073	0.069	**<0.001**	0.092	**<0.001**	0.023	0.081
0.35	74.80	**<0.001**	0.211	0.066	0.275	0.054	0.295	0.066	0.064	**<0.001**	0.085	**<0.001**	0.020	0.094
0.4	74.31	**<0.001**	0.185	0.061	0.244	0.051	0.262	0.060	0.059	**<0.001**	0.078	**<0.001**	0.018	0.106
0.45	73.13	**<0.001**	0.163	0.057	0.218	0.048	0.234	0.056	0.055	**<0.001**	0.071	**<0.001**	0.017	0.118
0.5	71.51	**<0.001**	0.145	0.052	0.195	0.045	0.210	0.052	0.050	**<0.001**	0.066	**<0.001**	0.015	0.127
***m* = 4**														
0.1	68.10	**<0.001**	0.510	0.144	0.647	0.121	0.688	0.158	0.136	**<0.001**	0.178	**<0.001**	0.042	0.133
0.15	70.46	**<0.001**	0.393	0.113	0.502	0.097	0.538	0.127	0.109	**<0.001**	0.145	**<0.001**	0.036	0.092
0.2	70.62	**<0.001**	0.321	0.093	0.411	0.081	0.441	0.103	0.090	**<0.001**	0.120	**<0.001**	0.031	0.076
0.25	71.45	**<0.001**	0.270	0.079	0.347	0.069	0.374	0.089	0.077	**<0.001**	0.104	**<0.001**	0.027	0.061
0.3	71.42	**<0.001**	0.233	0.070	0.300	0.061	0.324	0.077	0.067	**<0.001**	0.091	**<0.001**	0.024	0.060
0.35	72.06	**<0.001**	0.204	0.063	0.264	0.054	0.286	0.068	0.060	**<0.001**	0.082	**<0.001**	0.022	0.060
0.4	72	**<0.001**	0.180	0.057	0.235	0.049	0.254	0.061	0.055	**<0.001**	0.074	**<0.001**	0.019	0.068
0.45	71.81	**<0.001**	0.160	0.052	0.210	0.045	0.227	0.055	0.050	**<0.001**	0.068	**<0.001**	0.017	0.070
0.5	71.01	**<0.001**	0.143	0.048	0.189	0.042	0.205	0.050	0.046	**<0.001**	0.062	**<0.001**	0.016	0.076
***m* = 5**														
0.1	69.61	**<0.001**	0.486	0.137	0.616	0.119	0.665	0.159	0.130	**<0.001**	0.180	**<0.001**	0.050	**0.047**
0.15	69.65	**<0.001**	0.379	0.110	0.484	0.093	0.520	0.125	0.105	**<0.001**	0.141	**<0.001**	0.036	0.075
0.2	70.31	**<0.001**	0.311	0.092	0.400	0.079	0.429	0.101	0.088	**<0.001**	0.117	**<0.001**	0.029	0.092
0.25	70.65	**<0.001**	0.264	0.079	0.340	0.068	0.366	0.089	0.076	**<0.001**	0.102	**<0.001**	0.026	0.078
0.3	70.41	**<0.001**	0.227	0.070	0.294	0.060	0.318	0.078	0.067	**<0.001**	0.091	**<0.001**	0.024	0.062
0.35	70.12	**<0.001**	0.199	0.062	0.259	0.054	0.28	0.069	0.060	**<0.001**	0.081	**<0.001**	0.021	0.062
0.4	70.06	**<0.001**	0.176	0.056	0.230	0.049	0.249	0.061	0.054	**<0.001**	0.073	**<0.001**	0.019	0.070
0.45	69.37	**<0.001**	0.157	0.051	0.206	0.045	0.223	0.055	0.049	**<0.001**	0.066	**<0.001**	0.017	0.070
0.5	68.61	**<0.001**	0.141	0.047	0.185	0.041	0.201	0.050	0.045	**<0.001**	0.060	**<0.001**	0.016	0.076

F F-statistic, *SD* standard deviation, *MD* mean difference

Bold values indicate significant differences

Table S11 Sample entropy in the anterior-posterior direction as a function of *r* and *m* for a data length of *N*=1200 (i.e. 60-seconds): eyes closed – foam surface (CF)

	**One-way ANOVA**		**Summary statistics by group**						**Post-hoc**					
			**Young (Y)**		**Non-Fallers (NF)**		**Fallers (F)**		**NF - Y**		**F - Y**		**F - NF**	
r	**F**	**p-value**	**Mean**	**SD**	**Mean**	**SD**	**Mean**	**SD**	**MD**	**p-value**	**MD**	**p-value**	**MD**	**p-value**
***m* = 2**														
0.1	20.34	**<0.001**	0.667	0.142	0.738	0.111	0.764	0.148	0.071	**<0.001**	0.097	**<0.001**	0.026	0.435
0.15	23.51	**<0.001**	0.532	0.111	0.591	0.085	0.610	0.110	0.060	**<0.001**	0.079	**<0.001**	0.019	0.483
0.2	25.31	**<0.001**	0.440	0.100	0.497	0.079	0.512	0.097	0.057	**<0.001**	0.072	**<0.001**	0.015	0.585
0.25	25.36	**<0.001**	0.371	0.093	0.425	0.076	0.437	0.091	0.054	**<0.001**	0.066	**<0.001**	0.013	0.638
0.3	24.61	**<0.001**	0.315	0.086	0.365	0.073	0.377	0.087	0.050	**<0.001**	0.061	**<0.001**	0.012	0.653
0.35	23.83	**<0.001**	0.271	0.079	0.316	0.068	0.327	0.081	0.045	**<0.001**	0.056	**<0.001**	0.011	0.646
0.4	22.97	**<0.001**	0.235	0.072	0.276	0.064	0.286	0.075	0.041	**<0.001**	0.051	**<0.001**	0.010	0.617
0.45	22.15	**<0.001**	0.206	0.065	0.242	0.058	0.252	0.069	0.036	**<0.001**	0.046	**<0.001**	0.010	0.594
0.5	21.49	**<0.001**	0.182	0.059	0.214	0.053	0.223	0.062	0.032	**<0.001**	0.041	**<0.001**	0.009	0.572
***m* = 3**														
0.1	20.78	**<0.001**	0.615	0.158	0.697	0.124	0.717	0.161	0.082	**<0.001**	0.103	**<0.001**	0.020	0.667
0.15	21.93	**<0.001**	0.476	0.120	0.540	0.099	0.561	0.127	0.064	**<0.001**	0.085	**<0.001**	0.021	0.487
0.2	21.92	**<0.001**	0.394	0.097	0.445	0.080	0.463	0.102	0.051	**<0.001**	0.070	**<0.001**	0.018	0.423
0.25	22.99	**<0.001**	0.336	0.082	0.380	0.068	0.396	0.086	0.044	**<0.001**	0.060	**<0.001**	0.016	0.405
0.3	22.99	**<0.001**	0.292	0.073	0.332	0.061	0.345	0.075	0.040	**<0.001**	0.053	**<0.001**	0.013	0.466
0.35	23.28	**<0.001**	0.257	0.066	0.293	0.056	0.304	0.068	0.037	**<0.001**	0.047	**<0.001**	0.011	0.528
0.4	23.12	**<0.001**	0.228	0.061	0.261	0.052	0.271	0.062	0.034	**<0.001**	0.044	**<0.001**	0.010	0.534
0.45	23	**<0.001**	0.203	0.056	0.234	0.049	0.243	0.058	0.031	**<0.001**	0.040	**<0.001**	0.009	0.560
0.5	22.90	**<0.001**	0.182	0.052	0.211	0.046	0.219	0.054	0.029	**<0.001**	0.037	**<0.001**	0.008	0.570
***m* = 4**														
0.1	21.36	**<0.001**	0.592	0.151	0.671	0.119	0.693	0.159	0.079	**<0.001**	0.101	**<0.001**	0.022	0.604
0.15	22.49	**<0.001**	0.461	0.121	0.527	0.097	0.543	0.125	0.066	**<0.001**	0.082	**<0.001**	0.016	0.663
0.2	22.47	**<0.001**	0.380	0.101	0.434	0.083	0.450	0.104	0.055	**<0.001**	0.070	**<0.001**	0.015	0.581
0.25	23.09	**<0.001**	0.322	0.085	0.369	0.071	0.384	0.089	0.047	**<0.001**	0.061	**<0.001**	0.015	0.505
0.3	22.70	**<0.001**	0.280	0.074	0.320	0.063	0.333	0.078	0.040	**<0.001**	0.053	**<0.001**	0.013	0.491
0.35	22.55	**<0.001**	0.246	0.065	0.282	0.057	0.293	0.069	0.036	**<0.001**	0.047	**<0.001**	0.011	0.519
0.4	22.37	**<0.001**	0.219	0.059	0.251	0.051	0.261	0.062	0.032	**<0.001**	0.042	**<0.001**	0.010	0.500
0.45	22.18	**<0.001**	0.197	0.053	0.226	0.047	0.235	0.056	0.029	**<0.001**	0.038	**<0.001**	0.009	0.499
0.5	22.19	**<0.001**	0.177	0.049	0.204	0.044	0.212	0.051	0.027	**<0.001**	0.035	**<0.001**	0.009	0.495
***m* = 5**														
0.1	21.71	**<0.001**	0.560	0.143	0.636	0.116	0.660	0.156	0.076	**<0.001**	0.100	**<0.001**	0.024	0.514
0.15	22.43	**<0.001**	0.444	0.115	0.507	0.092	0.523	0.122	0.063	**<0.001**	0.079	**<0.001**	0.016	0.623
0.2	22.55	**<0.001**	0.368	0.098	0.422	0.080	0.436	0.102	0.054	**<0.001**	0.068	**<0.001**	0.014	0.609
0.25	23.24	**<0.001**	0.314	0.084	0.361	0.070	0.373	0.088	0.047	**<0.001**	0.059	**<0.001**	0.012	0.600
0.3	23.32	**<0.001**	0.273	0.074	0.314	0.062	0.325	0.077	0.041	**<0.001**	0.052	**<0.001**	0.011	0.587
0.35	23.09	**<0.001**	0.240	0.065	0.277	0.057	0.286	0.068	0.037	**<0.001**	0.046	**<0.001**	0.010	0.608
0.4	22.76	**<0.001**	0.214	0.058	0.247	0.052	0.255	0.061	0.033	**<0.001**	0.041	**<0.001**	0.009	0.597
0.45	22.27	**<0.001**	0.192	0.053	0.221	0.047	0.230	0.056	0.029	**<0.001**	0.038	**<0.001**	0.008	0.569
0.5	21.98	**<0.001**	0.174	0.048	0.200	0.044	0.208	0.051	0.026	**<0.001**	0.034	**<0.001**	0.008	0.543

F F-statistic, *SD* standard deviation, *MD* mean difference
Bold values indicate significant differences
